# Single‐Cell Analysis Identifies LYPD6B as a Tumor‐Intrinsic Candidate Associated With Immunotherapy Nonresponse in Breast Cancer

**DOI:** 10.1111/1759-7714.70311

**Published:** 2026-06-02

**Authors:** Yifei Wang, Haiwei Quan, Zhiguang Xu, Yixiang Wang, Zhibin Wang

**Affiliations:** ^1^ Center for Cancer Immunotherapy of Institute of Biomedicine and Biotechnology Shenzhen Institutes of Advanced Technology, Chinese Academy of Sciences Shenzhen Guangdong China; ^2^ Department of Biopharmaceutical Sciences, Faculty of Pharmaceutical Sciences Shenzhen University of Advanced Technology Shenzhen Guangdong China; ^3^ University of Chinese Academy of Sciences Beijing China; ^4^ Department of Biomedical Engineering Southern University of Science and Technology Shenzhen Guangdong China

**Keywords:** breast cancer, drug repurposing, immunotherapy resistance, PD‐1/PD‐L1, single‐cell transcriptomics

## Abstract

Immune checkpoint blockade (ICB) induces durable responses in a subset of breast cancer patients, yet many show limited benefit from anti‐PD‐1/PD‐L1 therapy. Understanding differences in the tumor microenvironment (TME) and tumor‐intrinsic immune evasion between responders (R) and nonresponders (NR) is key to improving outcomes. We integrated three public scRNA‐seq datasets from PD‐(L)1‐treated breast cancer (51 patients; 327022 cells) and analyzed paired pre‐/posttreatment samples. Compared to NR, R patients exhibited increased CD8^+^ T cell infiltration, enhanced interferon‐response activity, and myeloid/B‐cell remodeling. Pseudotime analysis showed T cells in R progressed from activation to cytotoxic differentiation and ultimately exhaustion, consistent with effective ICB response patterns. Through tumor‐intrinsic screening, we identified LYPD6B‐a membrane gene upregulated in NR cancer cells that suppresses antigen processing/presentation and IFNα/β signaling. Functional assays confirmed that LYPD6B ablation impairs proliferation, clonogenic growth, and induces apoptosis. Drug‐repurposing analyses revealed venetoclax binds LYPD6B and recapitulates its antiproliferative effects. Immunohistochemistry (IHC) and pan‐cancer analyses verified LYPD6B's tumor‐cell localization, association with immune infiltration, and checkpoint expression. Collectively, LYPD6B emerges as a tumor‐intrinsic mediator of immune evasion and PD‐(L)1 resistance, representing a promising target for combination immunotherapy in breast cancer.

## Graphical abstract

LYPD6B is a tumor‐intrinsic mediator of immune evasion and resistance to PD‐(L)1 therapy in breast cancer; targeting LYPD6B may improve immunotherapy efficacy.

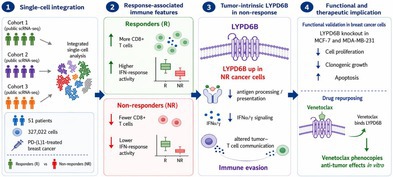

## 1 Introduction

ICB, particularly antibodies targeting the programmed cell death protein 1 (PD‐1) and its ligand PD‐L1, has transformed cancer therapy by reinvigorating antitumor T cell activity and producing durable responses across multiple solid tumors [1]. While breast cancer has historically been considered relatively low in immunogenicity, accumulating evidence demonstrates selected patients, especially those with triple‐negative breast cancer (TNBC) and other immunologically inflamed subtypes, have better responses [2, 3, 4, 5]. In metastatic TNBC, the addition of PD‐(L)1 blockade to chemotherapy has improved outcomes in patients with positive PD‐L1 expression, as shown in trials of atezolizumab plus nab‐paclitaxel and pembrolizumab plus chemotherapy [1, 6]. Nonetheless, response rates to ICB in breast cancer remain modest and vary substantially across patients, underscoring that durable benefit is limited to a minority and that primary resistance is frequent even among PD‐L1‐positive tumors [2, 3, 4, 5]. Mechanistically, clinical benefit from PD‐(L)1 blockade is generally associated with a pre‐existing T cell inflamed TME, featuring cytotoxic lymphocyte infiltration, interferon‐driven inflammatory programs, and intact antigen processing and presentation [4, 5, 7]. In contrast, resistance may arise from both extrinsic mechanisms, such as suppressive myeloid or stromal niches, and tumor‐intrinsic programs that attenuate immune recognition or effector function [4, 5, 8, 9].

Tumor‐intrinsic perturbations of IFN‐γ responsiveness and antigen presentation are particularly relevant because they can uncouple T cell attack from tumor cell susceptibility [10, 11, 12]. Clinical and experimental studies have linked acquired resistance to PD‐1 blockade to loss‐of‐function alterations in the IFN signaling pathway (e.g., JAK1, JAK2, and defects in antigen presentation components), thereby reducing IFN‐γ responses and MHC Class I surface expression [10, 11, 13]. Additional evidence indicates that alterations in JAK1 and JAK2 may also contribute to primary resistance by constraining IFNγ‐induced adaptive programs relevant to PD‐(L)1 blockade [10, 11]. Together, these findings motivate an integrated view of ICB resistance in which immune ecological states are causally intertwined with cancer cell‐intrinsic immune evasion programs [9, 12].

A major challenge in breast cancer is that these immune‐tumor couplings are difficult to disentangle with bulk profiling, which averages signals across cells including malignant, immune, and stromal compartments [14, 15, 16]. Single‐cell RNA sequencing (scRNA‐seq) enables quantitative profiling of cell‐type composition, functional states, and pathway programs at cellular resolution, facilitating direct comparisons of immune activation, interferon responses, and differentiation trajectories between clinical R and NR [14, 17, 18]. However, despite the growing availability of scRNA‐seq data for PD‐(L)1‐treated breast cancer, systematic multicohort evidence linking immune ecological characteristics, cancer‐intrinsic drivers of antigen presentation/IFN dysregulation, and the identification of druggable targets remains lacking [14, 19].

In this study, we performed integrated cross‐cohort analysis of scRNA‐seq data from multiple independent cohorts of PD‐(L)1‐treated breast cancer patients, which were strictly stratified into R group and NR group based on standard clinical response criteria. As a core approach to unravel the key mechanisms underlying ICB efficacy, our study systematically compared immune cell subset composition, their functional activation states, and key signaling pathway activities between R group and NR group at single‐cell resolution. Our goal was to gain precise insights into how immune activation is regulated in responsive versus nonresponsive tumors and lay a solid groundwork for identifying novel, functionally relevant therapeutic targets.

In addition to immune‐extrinsic factors, identifying tumor‐intrinsic drivers of ICB resistance is essential for improving treatment outcomes in breast cancer, which is another key focus of our study. We prioritized candidate molecules that modulate antigen processing/presentation pathways (including MHC Class I molecules and antigen‐processing enzymes) and IFNα/γ‐related signaling in malignant breast cancer cells, as perturbations in these pathways are closely linked to immune evasion and ICB resistance [20, 21, 22]. Through differential expression analysis of scRNA‐seq data and subsequent in vitro validation experiments, we identified LYPD6B as a potential tumor‐intrinsic mediator of ICB nonresponse. LYPD6B encodes a GPI‐anchored surface protein with a LU domain [20]. This is a family of molecules implicated in membrane signaling regulation and cell–cell interactions [20, 23], and recent preclinical studies have further linked LYPD6B to immunoregulation by limiting CD8^+^ T cell activation, suggesting its potential role in ICB resistance [24].

We further demonstrated that loss of LYPD6B suppresses breast cancer cell proliferation and clonogenic growth while increasing apoptosis. In parallel, we pursued a drug‐repurposing strategy and nominated venetoclax as a repurposing candidate for targeting LYPD6B. Together, these results establish a cross‐cohort framework that links response‐associated immune ecological features to a tumor cell‐intrinsic, membrane‐associated candidate mediator of immune evasion, and provide a rationale for exploring combination strategies integrating PD‐(L)1 blockade with LYPD6B‐directed interventions.

## 2 Materials and Methods

### 2.1 Data Sources and Group Definitions

Three publicly available scRNA‐seq cohorts of PD‐(L)1‐treated breast cancer were included (EGAS00001004809, NCT02999477, and GSE169246), comprising 51 patients and 327 022 cells. Paired pretreatment (Pre) and posttreatment (Post) tumor samples were available for each patient. Response status was defined as responder (R) or nonresponder (NR) according to the clinical outcome criteria reported in the original studies. Based on the clinical efficacy evaluations from the three datasets, patients assessed as complete response and partial response were classified as Rs, while those assessed as stable disease and progressive disease were classified as NRs. Downstream analyses comprised (i) R versus NR comparisons and (ii) four‐group comparisons by response and timepoint (Pre‐R, Post‐R, Pre‐NR, and Post‐NR) to assess treatment‐associated changes.

### 2.2 scRNA‐Seq Preprocessing and Annotation

Quality control, filtering, normalization, and highly variable gene selection were performed for each cohort separately. Datasets were then integrated with batch‐effect correction. We applied the Robust Principal Component Analysis (RPCA) [25] integration workflow from Seurat v4.0. The specific steps are as follows: based on the common highly variable genes from the three datasets, we performed independent PCA for each dataset. Subsequently, integration anchors were identified in the PCA space using the FindIntegrationAnchors function (parameters: reduction = “rpca,” dims = 1:30, k.anchor = 5), and finally, an integrated expression matrix was generated via the IntegrateDatafunction. Standardization and PCA dimensionality reduction were performed on the integrated data. We selected the first 20 principal components for downstream analysis. Cell clustering was performed using the FindNeighbors and FindClusters functions, with the clustering resolution parameter set to 0.5. Dimensionality reduction was conducted by principal component analysis, followed by clustering and UMAP visualization in the integrated space. Major cell populations, including malignant cells, T cells, B cells, myeloid cells, and endothelial/stromal cells, were annotated using canonical marker genes, and annotations were verified with dot plots and feature plots.

### 2.3 Immune IFN Response and Pathway Scoring

The IFN response module score was calculated for immune cells (or specific immune cell subsets). The average or median score per patient was used as the patient's immune IFN response indicator. As module scores are typically centralized/standardized relative to the entire background, values can be negative; a negative value does not indicate negative pathway activity but rather represents a level below the overall dataset average.

### 2.4 T Cell Reclustering and Pseudotime Analysis

Cancer infiltrating T cells were extracted and reclustered to define functional state subsets for CD4^+^ and CD8^+^ T cells. Using the Monocle3 package, pseudotime analysis was applied to construct state transition trajectories for T cells, and the dynamic changes of key genes along the pseudotime were assessed. Cells were then projected onto the pseudotime space based on their R/NR grouping to compare the distribution of the two groups across different trajectory stages and differences in key gene programs.

### 2.5 Differential Analysis

Differential expression analysis (R groups vs. NR groups) was performed on the integrated cancer cells, followed by gene set enrichment analysis (GSEA). GSEA was conducted in the R environment using the clusterProfiler package (version 4.6.0). The gene sets were obtained from the Molecular Signatures Database (MSigDB) [26] version v2023.2, specifically including the Hallmark [27] and C1–C8 subsets. All gene sets were downloaded online and imported via the msigdbr package (version 7.5.1). Candidate target screening followed a four‐tier prioritization: (1) cancer cell‐enriched expression (significantly higher than in immune/stromal cells); (2) upregulated in NR cells; (3) coupling to immune evasion phenotypes (downregulation of MHC‐I antigen presentation and/or IFN response); and (4) predicted cell‐surface/membrane accessibility for therapeutic tractability. The ligand–receptor interactions for tumor‐T cell communication were analyzed using the CellChatDB database [28]. The database version corresponds to the one integrated with the CellChat R package (v1.5.0).

### 2.6 Molecular Docking, Molecular Dynamics, and Efficacy Validation

In the absence of specific small‐molecule inhibitors for LYPD6B, a drug repurposing strategy was employed. A library of 2980 drug molecules from the marketed drug database DrugBank was screened using molecular docking with AutoDock Vina software47 [29]. The docking box center was set at the center of the target protein's active pocket (calculated via the PrankWeb server), with box dimensions of 35 Å × 35 Å × 35 Å, exhaustiveness set to 8, and the number of output binding conformations set to 10. We used the docking score (binding free energy Δ*G*) as the preliminary screening criterion. Compounds with Δ*G* < −10.0 kcal/mol were considered as potential high‐affinity candidates and proceeded to the next stage of analysis. A 50 ns molecular dynamics (MD) simulation was performed on the LYPD6B (Protein PDB ID: 6ZSO) complex using the AMBER 18 software [30]. The protein and venetoclax were modeled using the ff19SB [31] and GAFF [32] force fields, respectively, with the TIP3P water model. The simulation involved three stages: initial 5000 steps of energy minimization to eliminate structural clashes; subsequent heating from 0 to 300 K under gradually weakening restraints on backbone atoms (NVT/NPT ensembles, total 700 ps); finally, a 50 ns production NPT simulation under physiological conditions (300 K, 1 bar) with all restraints removed, using Langevin temperature control and Monte Carlo pressure control, a 2 fs timestep, and saving trajectories every 10 ps for subsequent binding mode and stability analysis. When the overall RMSD of the complex fluctuated by less than 2 Å during the final 20 ns of the simulation, we considered the system to have reached equilibrium, and its conformation was suitable for analysis. Dose–response experiments were conducted in MCF‐7 and MDA‐MB‐231 cells to calculate IC50 values, and efficacy was further validated using clonogenic and apoptosis assays.

### 2.7 Tissue and Pan‐Cancer External Validation

Publicly available immunohistochemistry images from the Human Protein Atlas (HPA) were used to observe LYPD6B localization in normal breast and breast cancer tissues, supplemented by quantitative information in Tables S2 and S3 summarizing staining intensity and cellular source. These images were also reviewed by the pathologist to confirm cell‐specific expression. The Tumor ImmunoEstimation Resource 2.0 (TIMER 2.0) website and Gene Expression Profiling Interactive Analysis 2 (GEPIA2) online platform were used for analyzing LYPD6B gene expression across various cancers. During the analysis, each expression value was transformed using the log2(*x* + 1) function, and the Benjamini–Hochberg method was applied to adjust the *p*‐values to control the false discovery rate (FDR) [33, 34].

### 2.8 In Vitro Experiments

#### 2.8.1 Cell Culture

MCF10A, MCF‐7, and MDA‐MB‐231 cell lines were obtained from ATCC and cultured according to the manufacturer's instructions. Cells were seeded in six‐well plates at a density of 1 × 10^6^ cells per well and allowed to adhere for 24 h before subsequent experiments.

#### 2.8.2 CRISPR‐cas9‐Mediated Gene Knockout

LYPD6B knockout cell lines were generated using a lentiviral CRISPR‐cas9 system. sgRNA oligonucleotides targeting LYPD6B were annealed and ligated into BsmBI‐digested lentiCRISPR v2 (Addgene #52961). A nontargeting sgRNA served as the negative control. Lentivirus was packaged in 293T cells by cotransfection of lentiCRISPR v2 with psPAX2 and pMD2.G using polyethyleneimine (PEI). Viral supernatants were harvested at 48 h post‐transfection, filtered, and used to transduce target cells for 24 h. Cells were subsequently collected for downstream validation, including quantitative PCR. The sgRNA sequences targeting LYPD6B (5′–3′) were 5′‐CTCTCGACCGTAAGTAGTGG‐3′ and 5′‐CTCGACCGTAAGTAGTGGTG‐3′.

#### 2.8.3 Drug Treatment and Phenotypic Assays

For pharmacological validation, cells were treated with venetoclax (VCX; MCE, HY‐15531) at the indicated concentrations. Dose–response assays were performed to generate cell viability curves and estimate IC_50_ values by nonlinear regression (MCF‐7: ~28.97 μM; MDA‐MB‐231: ~53.19 μM). For functional assays, venetoclax was applied at 10 μM (MCF‐7) and 20 μM (MDA‐MB‐231) for apoptosis analysis, and at 30 μM (MCF‐7) and 50 μM (MDA‐MB‐231) for colony formation assays. Apoptosis was assessed by Annexin V/PI staining followed by flow cytometry, and clonogenic capacity was evaluated using colony formation assays.

#### 2.8.4 Flow Cytometric Assay for Apoptosis

Cells for staining were washed with PBS and resuspended in Annexin‐binding buffer, followed by double staining with FITC‐conjugated Annexin V and PI (BD Pharmingen, Cat. No. 556547) in accordance with the manufacturer's instructions. Samples were acquired on a NovoCyte Advanteon VBR flow cytometer (Agilent), and the corresponding flow cytometry data were analyzed using Agilent NovoExpress Flow Cytometry Software.

### 2.9 Statistical Analysis

The detailed statistical methods for the bioinformatics section are described in the methods section of each part. For in vitro experiments, comparisons between two groups were performed using unpaired two‐tailed Student's *t*‐tests. Data are presented as mean ± standard deviation (SD) and were analyzed using GraphPad Prism software (version 8.0.2). A *p* value < 0.05 was considered statistically significant. Significance levels are indicated as *p* < 0.05, *p* < 0.01, and *p* < 0.001. Unless otherwise specified, all experiments were performed in triplicate.

## 3 Results

### 3.1 Cross‐Cohort Single‐Cell Integration Identifies Response‐Associated Immune Ecological Features in PD‐(L)1‐Treated Breast Cancer

**FIGURE 1:**
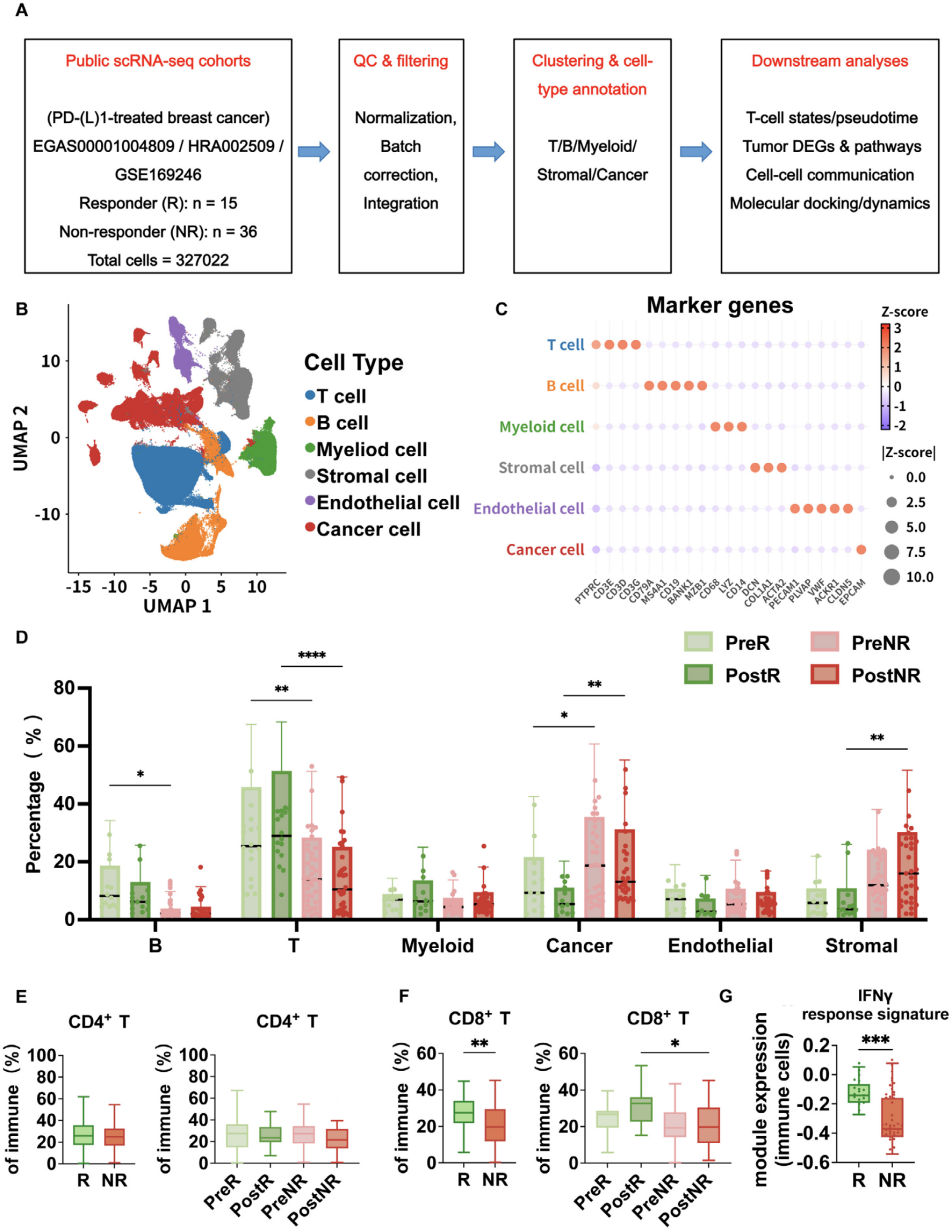
Multicohort single‐cell integration of PD‐(L)1‐treated breast cancer and patient‐level immune features. (A) Study design and cohort overview. Three independent scRNA‐seq cohorts of PD‐(L)1‐treated breast cancer were integrated. Samples were grouped by clinical response (R vs. NR) and by treatment time point (Pre vs. Post). (B) Integrated cellular atlas. UMAP of all cells showing major compartments, including cancer cells, T cells, B cells, myeloid cells, stromal cells, and endothelial cells. (C) Marker gene expression for cell‐type annotation. Dot plot of canonical marker genes used for annotation of the cell populations in (B). (D) Patient‐level cellular composition. Major cell‐type proportions were aggregated per patient. Comparisons are shown between R and NR and across four groups defined by response and time point (PreR, PostR, PreNR, PostNR). (E) CD4^+^ T cell infiltration. The CD4^+^ infiltration index was defined as the proportion of CD4^+^ T cells among total immune cells and summarized at the patient level. Comparisons are shown between R and NR and between Pre and Post within each response group. (F) CD8^+^ T cell infiltration. The CD8^+^ infiltration index was defined as the proportion of CD8^+^ T cells among total immune cells and summarized at the patient level. Comparisons are shown between R and NR and between Pre and Post within each response group. (G) IFN‐γ response signature. IFN‐γ module scores were computed in immune cells (or indicated subsets) and aggregated per patient. Comparisons are shown between R and NR. Statistical results of mean ± SD were shown; ns, indicates nonsignificant; *p < 0.05, **p < 0.01 (two‐tailed unpaired *t*‐test).

Three independent scRNA‐seq cohorts of PD‐(L)1‐treated breast cancer were used to establish a harmonized cross‐cohort analytical framework. Patients were stratified by clinical outcome into R (*n* = 15) and NR (*n* = 36) (Figure 1A). In the integrated whole‐cell UMAP representation, major cell populations formed well‐defined clusters, and cell‐type annotations were supported by canonical marker gene expression (Figure 1B,C). The distribution of R group and NR group cells in UMAP space, as well as marker‐based feature plots for all cells, are shown in Figures S1 and S2. We compared the batch‐colored PCA plot after integration. The results show that cells from different batches are well mixed in the low‐dimensional space after integration, with no clear batch separation. The corresponding validation plots are provided in Figure S9.

Aggregation of major cell‐type proportions at the patient level revealed differences in tumor and immune composition between groups. The calculation of cell proportions and inter‐group comparisons were performed at the patient level. Specifically, the frequency of each cell type was first calculated for each patient sample, and these frequency data were then used for inter‐group comparisons and correlation analyses. R group exhibited a higher overall proportion of T cells, whereas NR group showed a relative enrichment of malignant cells. Stromal compartments also displayed group‐associated trends (Figure 1D). Further analysis of T‐cell subsets showed that this increase was mainly reflected by higher CD8^+^ T cell infiltration in the R group, whereas CD4^+^ T cell proportions showed no obvious difference between the two groups (Figure 1E,F). Further supporting this immune‐activated phenotype, immune cells from the R group displayed significantly elevated interferon‐response module scores (Figure 1G), indicative of a more interferon‐associated, immune‐activated state. Collectively, these findings delineate immune ecological features associated with response, characterized by increased CD8^+^ T cell infiltration and enhanced interferon activity, whereas nonresponse is associated with a comparatively tumor‐enriched and less immune‐inflamed microenvironment.

### 3.2 A Continuous Spectrum of T Cell States Underlies Response‐Associated Activation and Exhaustion Dynamics

**FIGURE 2:**
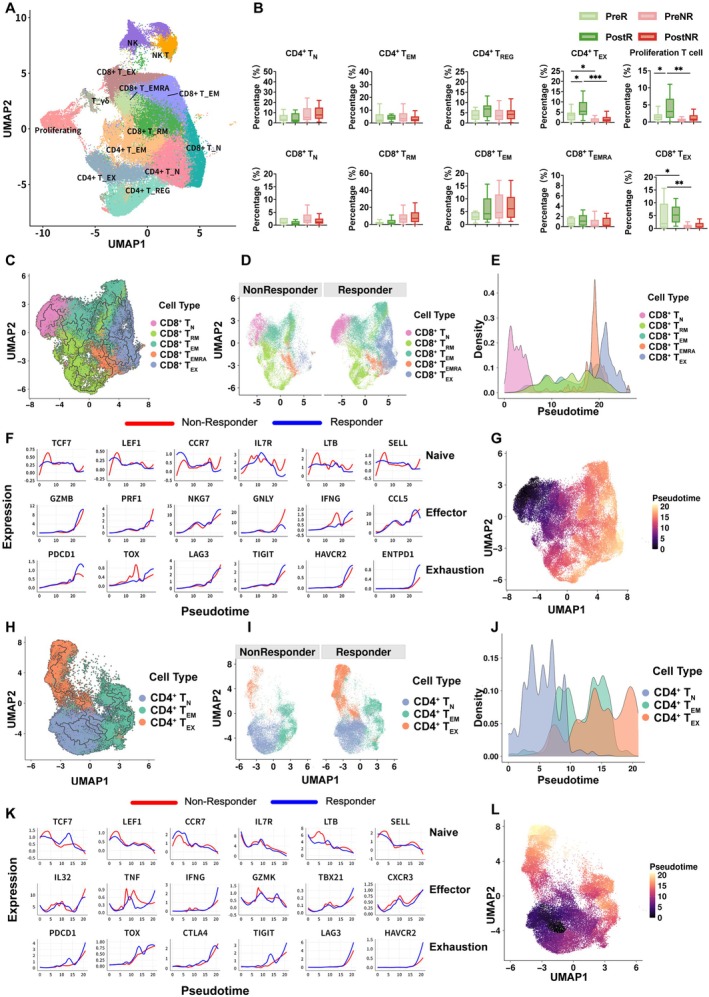
Distribution and dynamic features of cancer infiltrating T cell states. (A) Integrated atlas of cancer infiltrating T cells. Cancer infiltrating T cells from all cohorts were re clustered and visualized by UMAP. (B) Patient‐level comparison of T cell subset composition. The proportions of T cell subsets were aggregated per patient and compared between R and NR groups and/or across response‐by‐timepoint groups (PreR, PostR, PreNR, PostNR). Data are shown as box plots (median, interquartile range, whiskers at 1.5× interquartile range). (C) Pseudotime analysis of CD8^+^ T cells. Pseudotime inference was performed on CD8^+^ T cells to reconstruct a continuous state trajectory and visualized on the low‐dimensional embedding. (D) CD8^+^ T cells stratified by clinical response on the pseudotime embedding. CD8^+^ T cells were displayed on the pseudotime embedding and colored by response group (R vs. NR). (E) Density distribution of CD8^+^ T cells along pseudotime. Density plots show the distribution of CD8^+^ T cells across pseudotime. (F) Gene expression dynamics along CD8 pseudotime. Expression of representative marker genes was plotted along CD8 pseudotime for R and NR groups. (G) Pseudotime‐colored UMAP of CD8^+^ T cells. CD8^+^ T cells are displayed on the UMAP embedding and colored according to inferred pseudotime values. (H) Pseudotime analysis of CD4^+^ T cells. Pseudotime inference was performed on CD4^+^ T cells to reconstruct a continuous state trajectory and visualized on the low‐dimensional embedding. (I) CD4^+^ T cells stratified by clinical response on the pseudotime embedding. CD4^+^ T cells were displayed on the pseudotime embedding and colored by response group (R vs. NR). (J) Density distribution of CD4^+^ T cells along pseudotime. Density plots show the distribution of CD4^+^ T cells across pseudotime. (K) Gene expression dynamics along CD4^+^ pseudotime. Expression of representative marker genes was plotted along CD4 pseudotime for R and NR groups. (L) Pseudotime‐colored UMAP of CD4^+^ T cells. CD4^+^ T cells are displayed on the UMAP embedding and colored according to inferred pseudotime values. In (B), statistical results of mean ± SD were shown; ns, nonsignificant; *p < 0.05, **p < 0.01 (two‐tailed unpaired *t*‐test).

To further characterize the cellular basis of response‐associated immune differences, we reclustered tumor‐infiltrating T cells and identified distinct functional states within CD4^+^ and CD8^+^ T cell compartments (Figure 2A). In the UMAP representation, T cell subsets segregated according to established marker gene expression, which was further supported by heatmap visualization of functional and lineage‐associated genes (Figures S3–S5). Patient‐level analysis of subset proportions revealed systematic differences in T cell composition across response groups and treatment time points (Figure 2B), indicating that therapeutic response is associated with not only T cell abundance but also the distribution of functional T cell states.

Within the CD8^+^ T cell compartment, pseudotime trajectory analysis delineated a continuous progression from naive‐like states toward cytotoxic effector programs and subsequently toward an exhausted, negative‐feedback state (Figure 2C,G). Projection of cells by response status demonstrated distinct distribution patterns along the trajectory between R group and NR group (Figure 2D), which were further reflected in cell density profiles (Figure 2E). Consistent with this trajectory, naive‐associated genes (TCF7, LEF1, CCR7, and IL7R) were preferentially expressed at early pseudotime points and gradually declined, whereas cytotoxic effector genes (GZMB, GZMK, IFNG, and TBX21) and inhibitory or exhaustion‐associated receptors (PDCD1, TOX, CTLA4, TIGIT, LAG3, and HAVCR2) increased toward later stages (Figure 2F). Notably, R group exhibited greater dynamic induction of cytotoxic and interferon‐related programs, accompanied by increased expression of exhaustion‐associated markers, consistent with activation‐associated negative feedback following immune checkpoint blockade.

A similar continuum was observed within the CD4^+^ T cell compartment, spanning naive, effector, exhausted, and immunoregulatory (Treg‐associated) states (Figure 2H,L). Differences between R and NR were evident in both trajectory projections and density distributions (Figure 2I,J). Pseudotime‐dependent expression of key CD4^+^ T cell genes further indicated concurrent immune activation and immunoregulatory programs in R (Figure 2K), providing a cellular context for the increased representation of exhausted and regulatory CD4^+^ T cell states observed in this group.

### 3.3 Cancer Cell‐Intrinsic Screening Identifies LYPD6B for Impaired Antigen Presentation and Interferon Signaling

Based on the response‐associated immune differences in Figures 1 and 2, we next investigated cancer cell‐intrinsic determinants potentially contributing to immune evasion in the NR group. Single‐cell copy number variation analysis was performed using inferCNV (https://github.com/broadinstitute/inferCNV; v1.2.1), with T cells serving as the reference population to identify malignant cells (Figure S6). Cancer cells were subsequently extracted for downstream analyses.

**FIGURE 3:**
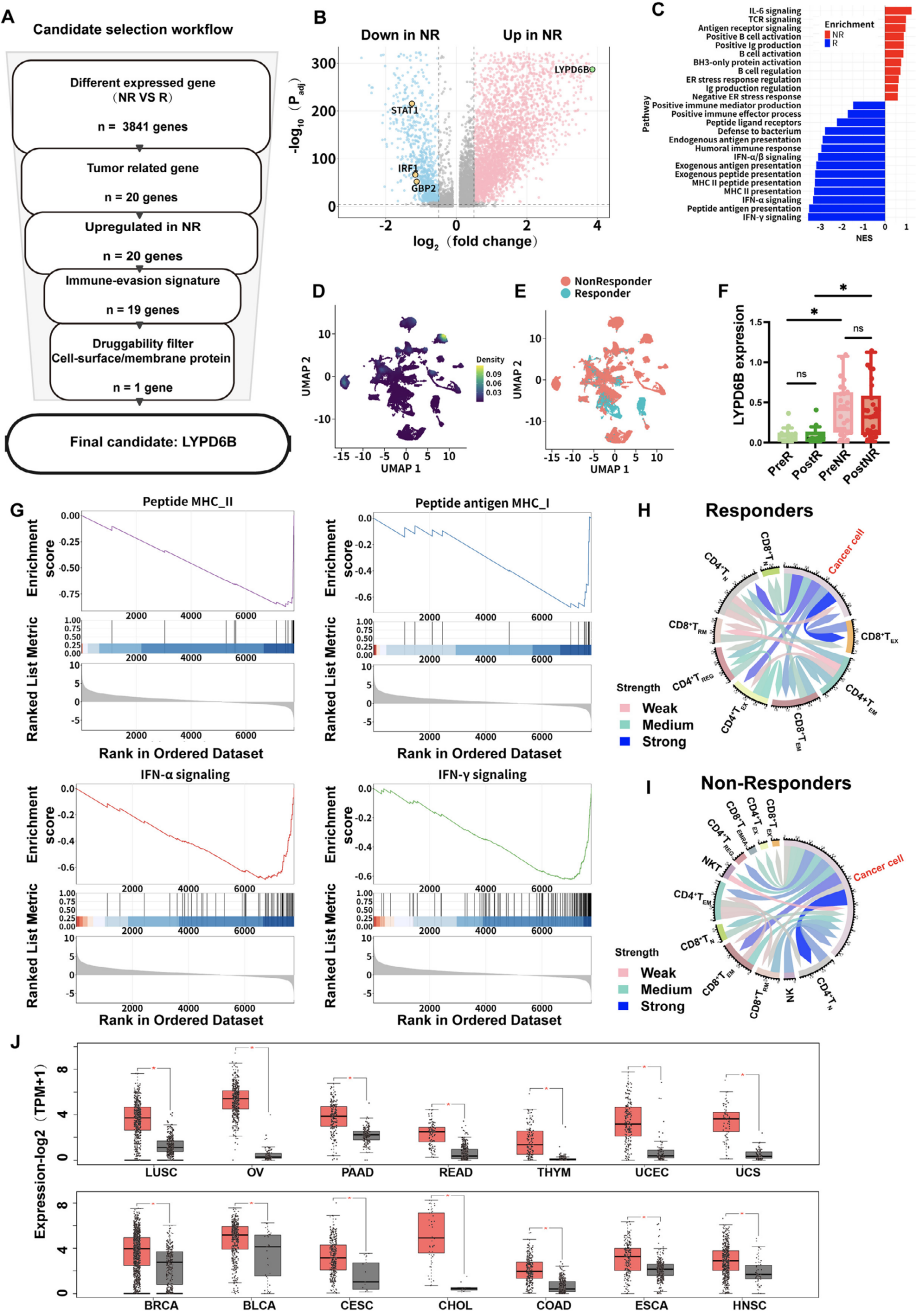
Cancer cell intrinsic candidate screening and immune interaction analysis. (A) Candidate screening workflow. Schematic overview of the stepwise framework used to identify cancer cell intrinsic candidates associated with response to PD‐(L)1 therapy, including differential expression analysis, immune evasion‐related filtering, and membrane or cell‐surface prioritization. (B) Differential expression analysis of cancer cells. Volcano plot showing genes differentially expressed between NR and R in cancer cells. The *x*‐axis represents the log2 fold‐change (log2FC), and the y‐axis represents the ‐log10 adjusted *p*‐value. Key candidate genes, including LYPD6B, are highlighted. (C) Pathway enrichment analysis of NR‐associated cancer cell genes. Bar plots showing enriched pathways derived from genes downregulated or upregulated in NR cancer cells. (D) LYPD6B expression density in cancer cells. UMAP of cancer cells colored by LYPD6B expression density across the integrated cohorts. (E) Distribution of cancer cells by response status. UMAP of cancer cells colored by clinical response group (R vs. NR). (F) Comparison of LYPD6B expression across response groups and time points. LYPD6B expression in cancer cells summarized at the patient level across PreR, PostR, PreNR, and PostNR groups. (G) GSEA showing negative enrichment of antigen presentation and interferon‐related pathways in NR cancer cells compared with R cancer cells. (H) Cancer cell‐T cell communication network in R. Chord diagram illustrating inferred ligand‐receptor interactions between cancer cells and T cell subsets in the R group. (I) Cancer cell‐T cell communication network in NR. Chord diagram illustrating inferred ligand‐receptor interactions between cancer cells and T cell subsets in the NR group. (J) Pan‐cancer expression of LYPD6B. Box plots showing LYPD6B expression across multiple cancer types and corresponding normal tissues using public cancer transcriptomic datasets. In (F), statistical results of mean ± SD were shown; ns, nonsignificant; *p < 0.05, **p < 0.01 (two‐tailed unpaired *t*‐test).

Differential expression analysis comparing NR group and R group cancer cells was conducted, followed by a multi‐step candidate prioritization strategy (Figure 3A). Starting from NR‐associated cancer cell differentially expressed genes (DEGs), candidates were sequentially filtered based on cancer cell‐enriched expression, preferential upregulation in NR, concordance with immune‐evasion features characterized by reduced MHC Class I antigen presentation and interferon response, and predicted membrane or cell‐surface accessibility. We applied a multistep filtering workflow starting from 3841 tumor‐cell DEGs between NRs and Rs. First, we selected genes with tumor‐cell‐enriched expression (*n* = 20). Second, we retained only those upregulated in NR (*n* = 20). Third, we filtered for genes whose expression correlated with downregulation of MHC class I antigen presentation or interferon response modules (*n* = 19). Finally, we prioritized membrane‐ or cell‐surface‐associated proteins, yielding a single candidate gene: LYPD6B. In contrast to ranking genes solely by effect size, this strategy emphasized cancer cell specificity, immune phenotype relevance, and therapeutic tractability.

In the volcano plot, LYPD6B was located among genes significantly upregulated in the NR group cancer cells (Figure 3B). Pathway enrichment analysis further revealed that genes downregulated in the NR group cancer cells were significantly enriched for antigen processing and presentation pathways, including MHC Class I and II‐related terms, whereas upregulated genes were associated with inflammatory and immune‐related signaling pathways such as IL‐6 signaling (Figure 3C). These results indicate that NR cancer cells exhibit a transcriptional program characterized by impaired antigen presentation.

Visualization of cancer cells in UMAP space showed localized subpopulations with high LYPD6B expression (Figure 3D), and enrichment of LYPD6B expression in NR group samples was evident when cells were colored by response status (Figure 3E). In four‐group comparisons stratified by treatment time point and response status, LYPD6B expression was consistently higher in NR group samples, with the highest expression observed in Post‐NR group (Figure 3F), suggesting an association with resistant disease states.

GSEA further showed that antigen presentation‐related pathways, including MHC class I and MHC class II antigen presentation, as well as IFNα‐ and IFNγ‐response pathways, were negatively enriched in NR cancer cells compared with R cancer cells (Figure 3G). These results indicate coordinated attenuation of antigen presentation and interferon‐related programs in NR cancer cells. These findings complement the elevated IFN‐response activity observed in immune cells from the R group (Figure 1G) and support a cancer cell‐intrinsic contribution to differential immune activation.

To determine potential functional consequences for tumor‐immune interactions, we compared communication networks between cancer cells and T cell subsets in R group and NR group. Chord diagram analysis revealed marked differences in interaction strength and connectivity patterns between groups (Figure 3H,I), indicating the LYPD6B‐associated transcriptional state may be linked to differential immunotherapy outcomes through modulation of cancer‐T cell communication. Finally, analyses of independent transcriptomic datasets showed that LYPD6B expression was elevated in tumor tissues relative to normal tissues across multiple cancer types (Figure 3J), suggesting broader relevance beyond breast cancer.

### 3.4 LYPD6B Expression Is Required for Breast Cancer Cell Growth

**FIGURE 4:**
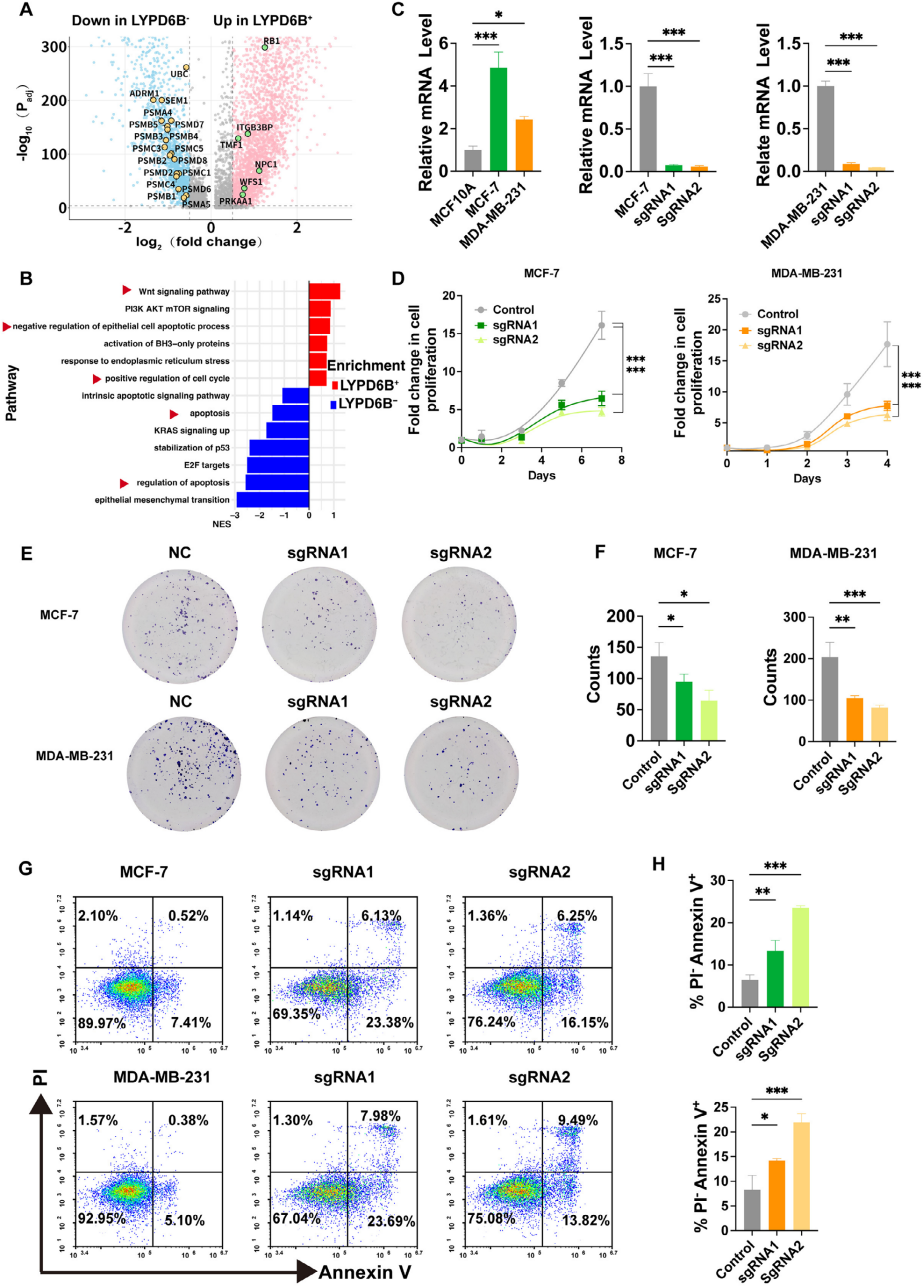
Functional validation of LYPD6B. (A) Differential expression analysis stratified by LYPD6B expression. Volcano plot showing genes differentially expressed between LYPD6B‐high and LYPD6B‐low cancer cell groups. The *x*‐axis indicates log2 fold change and the *y*‐axis indicates −log10 adjusted *p* value. (B) GSEA associated with LYPD6B expression. Bar plot showing normalized enrichment scores of representative pathways enriched in LYPD6B‐high and LYPD6B‐low groups. (C) LYPD6B expression and knockout validation in breast cell lines. Relative LYPD6B expression was measured in the nonmalignant breast epithelial cell line MCF10A and breast cancer cell lines MCF‐7 and MDA‐MB‐231. LYPD6B knockout efficiency was validated in MCF‐7 and MDA‐MB‐231 cells using two independent sgRNAs. (D) Cell proliferation assay. CCK‐8 assays showing the proliferation of control and LYPD6B‐knockout MCF‐7 and MDA‐MB‐231 cells. (E) Colony formation assay. Representative images of colony formation in MCF‐7 and MDA‐MB‐231 cells under control and LYPD6B knockout conditions. (F) Quantification of clonogenic capacity. Bar plots summarizing colony numbers from (F). (G) Flow cytometric analysis of apoptosis. Representative Annexin V/PI flow cytometry plots showing apoptotic cells in control and LYPD6B‐knockout MCF‐7 and MDA‐MB‐231 cells. (H) Quantification of apoptotic cells. Bar plots summarizing the proportion of apoptotic cells from (G). Data are presented as mean ± SD. ns, not significant; *p < 0.05, **p < 0.01 (two‐tailed unpaired *t*‐test).

To examine whether elevated LYPD6B expression is linked to specific cancer cell‐intrinsic programs, we performed differential expression analysis comparing LYPD6B‐high and LYPD6B‐low cancer cells. Volcano plot analysis revealed widespread transcriptional differences between the two groups (Figure 4A). Pathway enrichment analysis showed that genes associated with high LYPD6B expression were significantly enriched in proliferation‐ and survival‐related pathways, including Wnt signaling, PI3K–AKT–mTOR signaling, KRAS signaling, and E2F targets, as well as pathways related to apoptosis regulation and endoplasmic reticulum stress (Figure 4B). To investigate the molecular mechanism underlying LYPD6B‐mediated apoptosis regulation, we further analyzed the expression of genes associated with the “negative regulation of epithelial cell apoptotic process” pathway. As shown in Figure S10, multiple key antiapoptotic genes, including PBX1 and BCL2, were significantly upregulated in LYPD6B^+^ cancer cells. This finding provides direct transcriptomic evidence for the pro‐survival function of LYPD6B, suggesting that it may inhibit cell death by activating an intrinsic anti‐apoptotic program, thereby explaining the observed increase in apoptosis upon LYPD6B knockout.

At the cellular level, LYPD6B expression was lower in the nonmalignant breast epithelial cell line MCF10A but markedly higher in the breast cancer cell lines MCF‐7 and MDA‐MB‐231 (Figure 4C, left). To assess the functional role of LYPD6B, two independent sgRNAs were used to disrupt LYPD6B expression, resulting in a substantial reduction of LYPD6B protein levels in both cancer cell lines (Figure 4C, middle and right). LYPD6B knockout significantly suppressed cell proliferation in MCF‐7 and MDA‐MB‐231 cells (Figure 4D) and markedly reduced clonogenic capacity (Figure 4E,F).

Consistent with these growth‐inhibitory effects, flow cytometry revealed an increased proportion of early apoptotic cells following LYPD6B loss in both cell lines (Figure 4G,H). Together, these results demonstrate that LYPD6B expression is associated with pro‐survival transcriptional programs and that LYPD6B is functionally required for breast cancer cell proliferation and survival.

### 3.5 Drug Repurposing Identifies Venetoclax as a Candidate Modulator of LYPD6B

**FIGURE 5:**
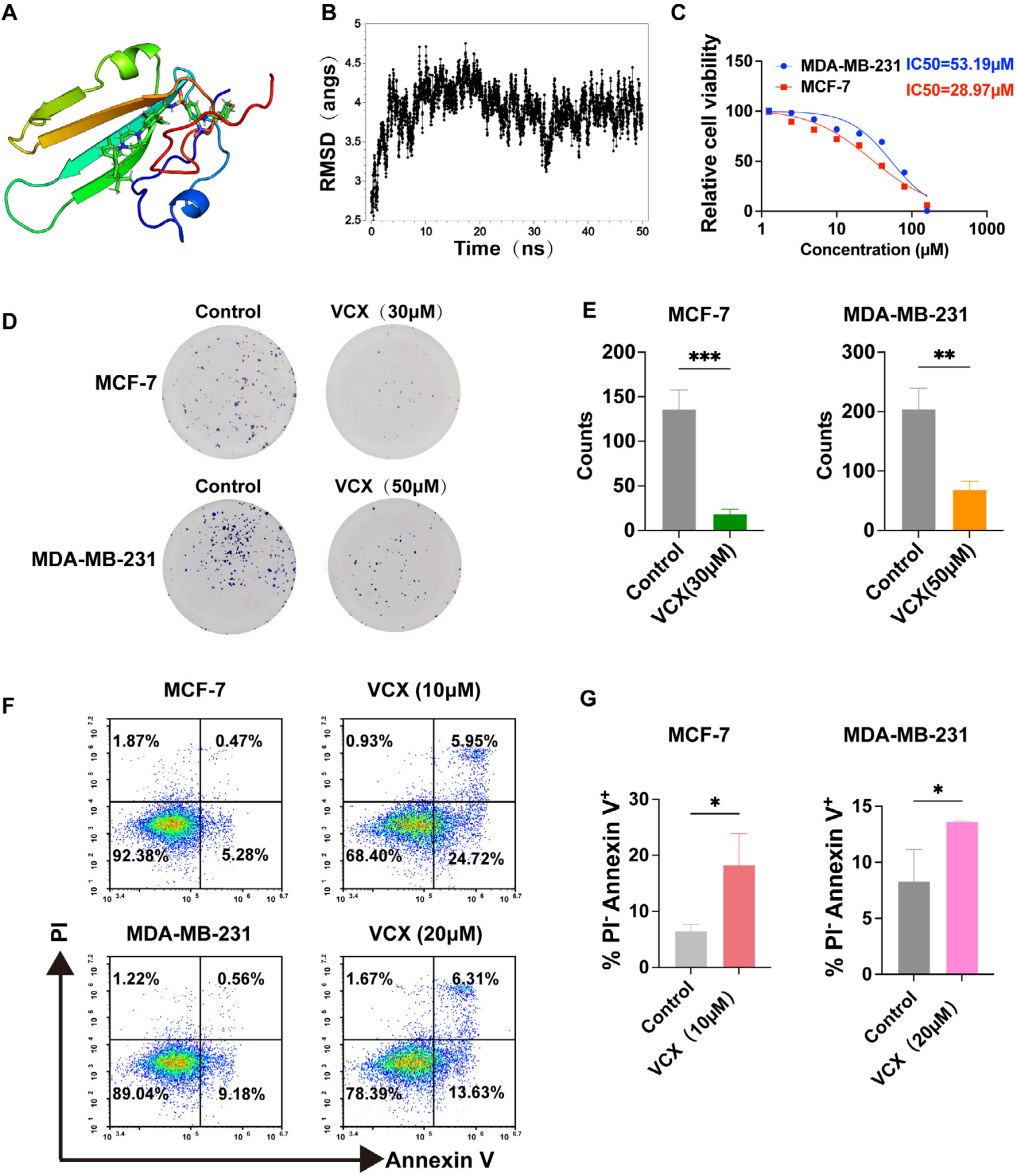
Structural modeling and functional assessment of venetoclax in relation to LYPD6B. (A) Structure of the LYPD6B‐venetoclax complex. The three‐dimensional structure of the complex formed between the LYPD6B protein and the small molecule VCX, obtained through molecular docking is presented. (B) RMSD analysis from molecular dynamics simulation. A 50 ns molecular dynamics simulation was performed on the LYPD6B‐VCX complex. The root‐mean‐square deviation (RMSD) trajectory over time is plotted to validate the structural stability of the complex. (C) Dose–response analysis of venetoclax in breast cancer cell lines. Cell viability curves and estimated IC_50_ values for venetoclax treatment in MCF‐7 and MDA‐MB‐231 cells. (D) Colony formation assay following venetoclax treatment. Representative images of colonies formed by MCF‐7 and MDA‐MB‐231 cells treated with venetoclax at the indicated concentrations. (E) Quantitative analysis of the colony formation assay. The results from (D) were quantitatively analyzed. Statistical results demonstrate that VCX treatment significantly reduced the number of colonies formed by both MCF‐7 and MDA‐MB‐231 cells. (F) Detection of venetoclax‐induced apoptosis. Apoptosis in MCF‐7 and MDA‐MB‐231 cells after VCX treatment was detected by flow cytometry. (G) Quantification of apoptotic cells. Summary of apoptotic cell fractions corresponding to (F). Data are presented as mean ± SD. Statistical significance was assessed using two‐tailed unpaired *t*‐tests. ns, not significant; *p < 0.05; **p < 0.01.

In the absence of specific small‐molecule inhibitors targeting LYPD6B, we applied a drug‐repurposing strategy to identify clinically available compounds with potential binding affinity for LYPD6B and performed in vitro evaluation. Next, we did molecular docking analysis to identify venetoclax as a top candidate capable of forming a stable complex with LYPD6B (Figure 5A). The Top 10 compounds ranked by docking score are listed in Table S4. To further determine complex stability, we performed a 50 ns MD simulation. The root‐mean‐square deviation (RMSD) trajectory showed an initial equilibration phase within the first ~5 ns, followed by stable fluctuations without progressive drift throughout the remainder of the simulation (Figure 5B), indicating a conformationally stable LYPD6B‐venetoclax complex. This result indicates that the LYPD6B‐venetoclax complex reached dynamic equilibrium within the 50 ns simulation time. Its overall three‐dimensional structure remained stable during this period. This verifies that the binding between venetoclax and the LYPD6B protein is stable. It also suggests that this drug molecule could be a potential inhibitor of LYPD6B. And we performed detailed binding site analysis on the equilibrated phase of the MD simulation (30–50 ns, 40 frames in total). Using a 3.5 Å distance cutoff, we calculated the contact occupancy of each protein residue with Venetoclax (LYPD6B ligand), defining residues with > 60% occupancy as key binding sites. The analysis identified 8 key binding residues that exhibited stable interaction patterns during the equilibrated simulation phase. THR88 showed the highest contact occupancy (97.5%), maintaining contact in 39/40 frames, indicating its critical role as a hydrogen bond donor/acceptor. LEU86 and ASN89 also demonstrated high stability (95% occupancy), participating in hydrophobic packing and hydrogen bonding, respectively. Together, these residues form a functionally complementary binding pocket comprising a hydrogen bond network (THR88, ASN89, THR34, SER75), hydrophobic interactions (LEU86), and structural stabilizing elements (HIE36, CYS82).

At the cellular level, venetoclax treatment reduced viability of MCF‐7 and MDA‐MB‐231 cells in a dose‐dependent manner, yielding IC50 values of 28.97 and 53.19 μM, respectively (Figure 5C). Consistent with these effects, venetoclax significantly decreased clonogenic growth in both cell lines in long‐term colony formation assays (Figure 5D,E). Flow cytometric analysis further demonstrated a reduction in the proportion of viable cells, accompanied by increased early apoptotic cells upon venetoclax treatment (Figure 5F,G). Although the precise binding interface and target specificity of venetoclax toward LYPD6B remain to be determined experimentally, these results provide initial evidence supporting venetoclax as a pharmacological candidate for probing LYPD6B‐associated biology. These results motivate further development of LYPD6B‐directed intervention strategies.

### 3.6 Cancer Cell‐Predominant Expression of LYPD6B Is Associated With Immune Infiltration and Checkpoint Profiles

**FIGURE 6:**
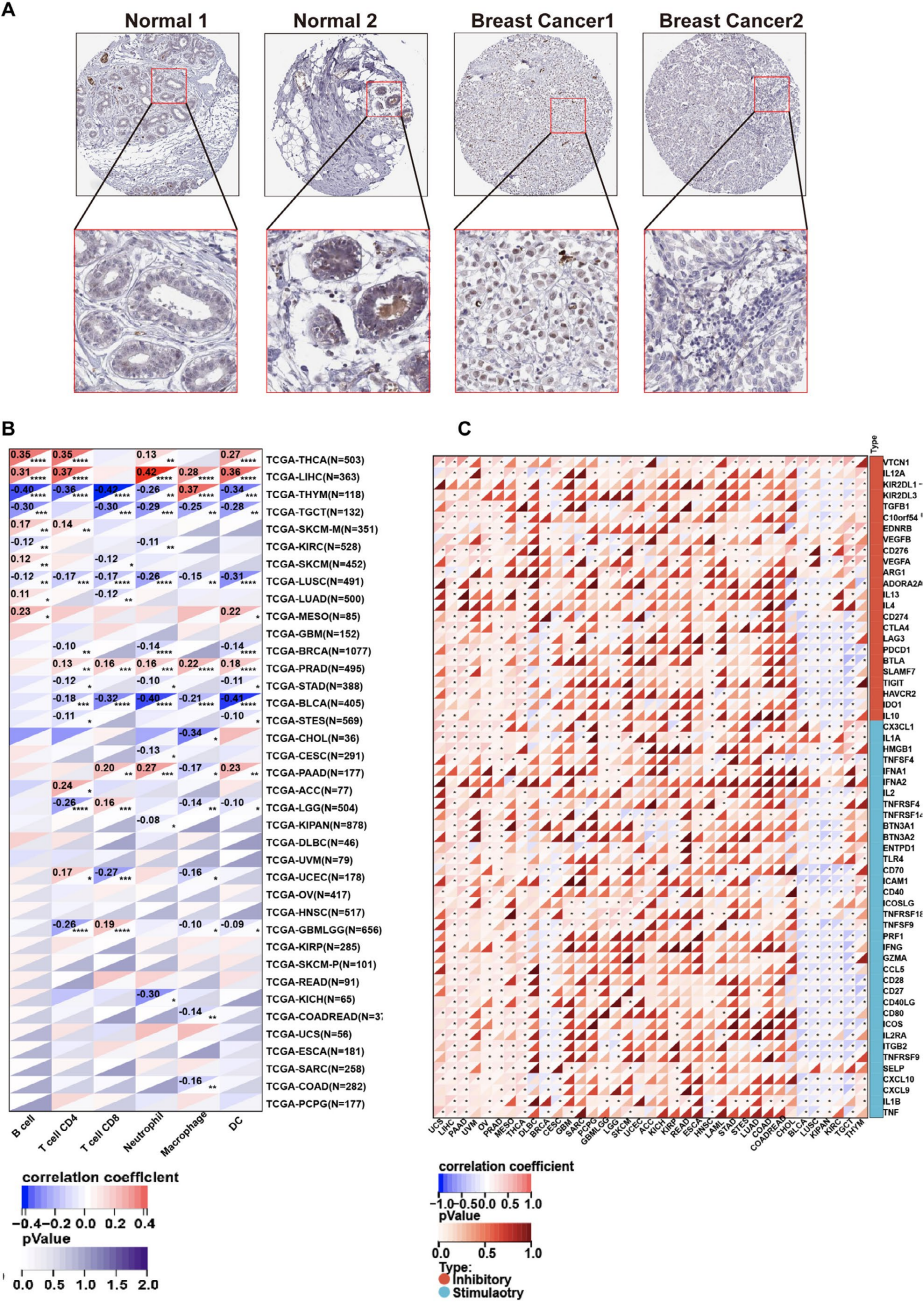
Tissue distribution of LYPD6B and pan‐cancer immune correlations. (A) Immunohistochemical localization of LYPD6B in breast tissue. Representative immunohistochemistry images from the HPA showing LYPD6B protein expression in normal breast tissue and breast cancer tissue. Enlarged regions illustrate staining patterns in epithelial and tumor areas. (B) Pan‐cancer correlations between LYPD6B expression and immune cell infiltration. Heatmap showing correlation coefficients between LYPD6B expression and estimated infiltration scores of immune cell types, including macrophages, NK cells, B cells, neutrophils, and dendritic cells, across multiple cancer types. Red indicates positive correlation and blue indicates negative correlation. (C) Pan‐cancer correlations between LYPD6B expression and immune checkpoint genes. Heatmap showing correlations between LYPD6B expression and inhibitory or stimulatory immune checkpoint gene expression across cancer types. Red indicates positive correlation and blue indicates negative correlation.

To determine the cellular localization of LYPD6B, we analyzed immunohistochemistry data from breast tissues available in the HPA. In normal breast tissue, LYPD6B staining was predominantly observed in glandular epithelial cells, whereas adipocytes and myoepithelial cells exhibited minimal or no staining. In breast cancer specimens, LYPD6B expression was primarily localized to malignant regions in most samples (Figure 6A). Consistent with these observations, LYPD6B was infrequently detected in cancer‐infiltrating immune cells, based on staining intensity, the proportion of positive cells, and subcellular localization across normal and tumor tissues (Tables S2 and S3).

To extend these observations to a broader cancer context, we performed pan‐cancer analyses using expression data from TCGA, TARGET, and GTEx in conjunction with re‐estimated immune infiltration scores. Correlation analysis demonstrated significant associations between LYPD6B expression and infiltration scores for multiple immune cell types, including T cells and macrophages, across several cancer types, with the direction of correlation varying by tumor type (Figure 6B). In TCGA‐BRCA, LYPD6B expression showed a negative correlation trend with T cell infiltration scores, consistent with the reduced immune infiltration observed in the NR group. In addition, LYPD6B expression exhibited broad correlation patterns with immune checkpoint‐related genes across cancer types (Figure 6C), situating LYPD6B within a broader immune regulatory landscape.

### 3.7 Response‐Associated Remodeling of Myeloid and B Cell Compartments

**FIGURE 7:**
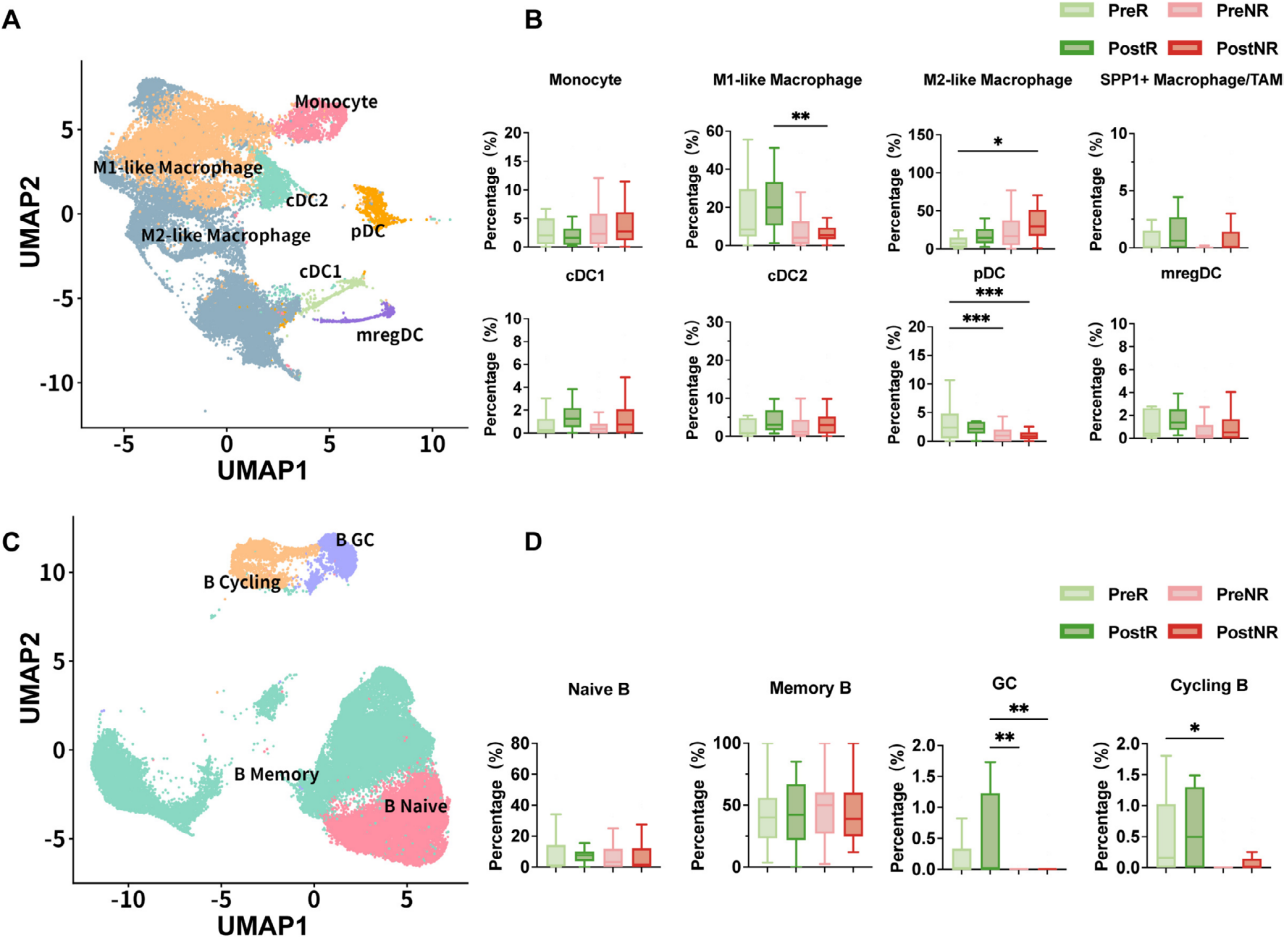
Dynamic changes in myeloid and B cell subsets before and after PD‐(L)1 treatment. (A) Integrated atlas of myeloid cell subsets. Cancer‐infiltrating myeloid cells from all cohorts were integrated and re‐clustered. UMAP visualization shows major myeloid subsets, including monocytes, M1‐like macrophages, M2‐like macrophages, SPP1^+^ TAMs, cDC1, cDC2, pDCs, and mregDCs. (B) Patient‐level comparison of myeloid cell subset proportions. The proportions of myeloid cell subsets were quantified per patient and compared across response and treatment groups (PreR, PostR, PreNR, PostNR). (C) Integrated atlas of B cell subsets. Cancer‐infiltrating B cells were re‐clustered, and UMAP visualization delineates major B cell subsets, including naive B cells, memory B cells, germinal center (GC) B cells, and cycling B cells. (D) Patient‐level comparison of B cell subset proportions. The proportions of B cell subsets were quantified per patient and compared across response and treatment groups (PreR, PostR, PreNR, PostNR). Data are presented as mean ± SD. ns, not significant; *p < 0.05, **p < 0.01 (two‐tailed unpaired *t*‐test).

Given the established roles of myeloid cells and B cells in shaping immune checkpoint blockade responses, we performed reclustering analyses of myeloid and B cell populations. UMAP visualization of myeloid cells identified multiple functional subsets, including monocytes, M1‐like and M2‐like macrophages, SPP1^+^ tumor‐associated macrophages (TAMs), conventional dendritic cells (cDC1 and cDC2), plasmacytoid dendritic cells (pDCs), and mature regulatory dendritic cells (mregDCs) (Figure 7A). Myeloid subsets were defined by canonical marker gene expression (Figure S8). Patient‐level comparison of myeloid subset proportions revealed differential enrichment patterns between R group and NR group. R group was enriched for immune‐activating subsets, including M1‐like macrophages and pDCs, whereas NRs showed a relative increase in M2‐like macrophages (Figure 7B), consistent with a more immune‐suppressive myeloid composition. Reclustering of B cells identified the subsets of naive, memory, germinal center (GC), and cycling B cell (Figure 7C), with corresponding marker gene expression shown in Figure S7. Quantitative comparison demonstrated that GC B cells and cycling B cells were significantly enriched in Rs relative to NRs (Figure 7D), indicating a response‐associated shift in B cell composition.

## 4 Discussion

This study integrated multiple publicly available single‐cell cohorts of PD‐(L)1‐treated breast cancer and quantified TME features at the patient level rather than solely at the cellular level, enabling a more robust cross‐cohort characterization of immune ecological differences between R group and NR group [5, 14, 35, 36]. Overall, tumors from R patients exhibited a more immune‐inflamed and immune‐activated phenotype, characterized by increased T cell infiltration and enhanced interferon‐related responses, whereas tumors from NR patients more frequently displayed features consistent with an immune‐cold or immune‐evasive state (Figure 1), this finding aligns with previous studies [4, 5, 36, 37]. These observations provide a rationale for subsequent mechanistic investigation and suggest that nonresponse is not simply attributable to reduced immune cell abundance, but instead may reflect tumor‐intrinsic programs that limit immune activation or immune maintenance [37].

Notably, although T‐cell exhaustion markers are often considered less abundant in responsive tumors [38, 39], our analysis revealed increased expression of inhibitory receptors (PDCD1, TOX, LAG3, TIGIT, HAVCR2) and enrichment of Treg populations in R tumors (Figure 2). Importantly, this phenotype should not be interpreted as terminal exhaustion or irreversible dysfunction. Rather, the coexistence of cytotoxic/activation‐associated features with inhibitory receptor expression suggests an activation‐associated, potentially reversible exhausted state, consistent with functionally engaged T cells under feedback inhibitory control [17, 38]. Recent studies suggest that ICB works by reducing feedback inhibition rather than eliminating exhausted T cells [40, 41]. Our findings are consistent with this view. T cells in NR tumors rarely enter this reversible exhaustion state. Instead, they remain in a functionally inactive state. This distinction highlights the critical role of T cell exhaustion plasticity in determining ICB response. It also provides a new perspective for understanding ICB therapy response heterogeneity.

We aimed to identify potential therapeutic targets. Thus, we did not only rank DEGs by their expression levels [21, 42]. Instead, we constructed a framework to select optimal candidate targets. We filtered candidates based on four key criteria: cancer cell specificity, higher expression in the NR group, association with immune evasion, and membrane localization (Figure 3A). Through this strategy, LYPD6B emerged as a leading candidate. LYPD6B was consistently upregulated in NR cancer cells (Figure 3D–F). Elevated LYPD6B expression was associated with reduced antigen presentation and attenuated IFN signaling (Figure 3C,G). Previous studies have identified these as key causes of ICB resistance [22, 42]. Our study is the first to reveal that LYPD6B expression correlates with tumor‐intrinsic immune evasion in breast cancer. We also analyzed ligand–receptor interactions between cells. The results showed that cancer cell‐intrinsic states alter the communication between cancer cells and T cells (Figure 3H,I). Recent studies suggest that altered intercellular signaling contributes to ICB resistance [43]. Our findings support this conclusion.

Functional validation demonstrated that LYPD6B is more highly expressed in breast cancer cell lines than in nonmalignant epithelial cells and that CRISPR‐mediated disruption of LYPD6B suppresses proliferation, reduces clonogenic capacity, and increases apoptosis (Figure 4). These findings support the biological relevance of LYPD6B in tumor cell‐intrinsic survival programs. Tissue‐level analyses further confirmed predominant localization of LYPD6B within malignant regions (Figure 6A; Tables S3 and S4), and pan‐cancer analyses extended these observations across multiple tumor types (Figure 6B,C) [37, 44], situating LYPD6B within a broader immune regulatory landscape.

To explore potential therapeutic strategies, we employed a drug repurposing approach and identified venetoclax as a candidate compound capable of binding to LYPD6B through molecular docking and dynamics simulations (Figure 5). In vitro treatment reduced proliferation and increased apoptosis in breast cancer cell lines. However, given the known activity of venetoclax against BCL2 [45, 46], off‐target effects cannot be excluded. This finding indicates the need for specific inhibitors against LYPD6B. Such specific inhibitors are currently lacking and represent a critical research gap [47].

In this study, integrative single‐cell analyses across multiple cohorts link LYPD6B expression to transcriptional features associated with immunotherapy nonresponse. Elevated LYPD6B expression correlates with reduced antigen presentation and interferon signaling signatures. In vitro experiments further show that LYPD6B promotes breast cancer cell proliferation and survival, supporting its role in tumor cell‐intrinsic programs. Defining the potential involvement of LYPD6B in immunotherapy resistance is therefore a central focus of this work. The mechanisms underlying this association are likely complex. LYPD6B may influence tumor‐intrinsic signaling, immune cell interactions, and tumor–immune communication within the microenvironment. Clarifying these processes will require further mechanistic investigation.

In summary, our study identifies LYPD6B as a novel tumor‐intrinsic factor associated with nonresponse to PD‐(L)1 therapy in breast cancer. LYPD6B expression is linked to transcriptional features consistent with known ICB resistance mechanisms [22, 42]. We further extend these observations across multiple cancer types. Collectively, these findings suggest that LYPD6B may represent a potential target for future combination strategies aimed at improving immunotherapy outcomes. In addition, a preliminary version of this study has been deposited as a preprint on the Lang‐TaoSha Preprint Server (DOI: https://doi.org/10.65215/LTSpreprints.2026.02.28.000146).

## Author Contributions

**Haiwei Quan:** writing – review and editing, software. **Yixiang Wang:** writing – review and editing. **Zhiguang Xu:** writing – review and editing, software. **Yifei Wang:** investigation, methodology, validation, writing – original draft, visualization, writing – review and editing, software, data curation, formal analysis, conceptualization, resources. **Zhibin Wang:** conceptualization, writing – original draft, writing – review and editing, funding acquisition, supervision, project administration, resources.

## Funding

This work was supported by the National Key Research and Development Program of China (Grant 2023YFA0915700) to Z.W.

## Ethics Statement

The patient data used in this study come from published, de‐identified datasets; in vitro cell experiments did not involve human/animal ethics approval.

## Conflicts of Interest

The authors declare no conflicts of interest.

## Supporting information


**Figure S1:** Distribution of responder versus nonresponder for all cells in UMAP space.
**Figure S2:** UMAP of all cells, colored by expression of canonical marker genes.
**Figure S3:** UMAP of T cells color‐coded for one marker gene per T cell phenotype.
**Figure S4:** Heatmap of functional marker gene expression across T cell subtypes.
**Figure S5:** Heatmap of marker gene expression across T cell subtypes.
**Figure S6:** CNV profile in cancer versus T cells assessed using InferCNV based on scRNA‐seq.
**Figure S7:** Heatmap of marker gene expression across B cell subtypes.
**Figure S8:** Heatmap of marker gene expression across myeloid cell subtypes.
**Figure S9:** The batch‐colored PCA plot after integration.
**Figure S10:** Heatmap of antiapoptosis related gene expression in LYPD6B^+^ versus LYPD6B^−^ clusters.
**Table S1:** Aboreviation and full name.
**Table S2:** Summarized pathological and quantification data of immunohistochemical analysis of TOX1 in normal breast.
**Table S3:** Summarized pathological and quantification data of immunohistochemical analysis of LYPD6B in breast cancer.
**Table S4:** Top 10 drug molecules ranked by docking score.

## Data Availability

The single‐cell transcriptomic data used in this study are from public databases (EGAS00001004809, NCT02999477, and GSE169246). Analysis code and key intermediate results can be provided upon submission according to journal requirements. The data that support the findings of this study are available from the corresponding author upon reasonable request.
