# Supplementary material for: Single‐Cell Analysis Identifies LYPD6B as a Tumor‐Intrinsic Candidate Associated With Immunotherapy Nonresponse in Breast Cancer
Source: Thorac Cancer. 2026 Jun 2;17(11):e70311. doi: 10.1111/1759-7714.70311 (PMC13240201; doi:10.1111/1759-7714.70311)
Supplement: Supplementary file 1 — Figure S1: Distribution of responder versus nonresponder for all cells in UMAP space. Figure S2: UMAP of all cells, colored by expression of canonical marker genes. Figure S3: UMAP of T cells color‐coded for one marker gene per T cell phenotype. Figure S4: Heatmap of functional marker gene expression across T cell subtypes. Figure S5: Heatmap of marker gene expression across T cell subtypes. Figure S6: CNV profile in cancer versus T cells assessed using InferCNV based on scRNA‐seq. Figure S7: Heatmap of marker gene expression across B cell subtypes. Figure S8: Heatmap of marker gene expression across myeloid cell subtypes. Figure S9: The batch‐colored PCA plot after integration. Figure S10: Heatmap of antiapoptosis related gene expression in LYPD6B+ versus LYPD6B− clusters. Table S1: Aboreviation and full name. Table S2: Summarized pathological and quantification data of immunohistochemical analysis of TOX1 in normal breast. Table S3: Summarized pathological and quantification data of immunohistochemical analysis of LYPD6B in breast cancer. Table S4: Top 10 drug molecules ranked by docking score. [file TCA-17-e70311-s001.docx]

**Single-cell analysis identifies LYPD6B as a tumor-intrinsic candidate associated with immunotherapy non-response in breast cancer**

Yifei Wang^1,2,3^, Haiwei Quan^2,4^, Zhiguang Xu^2^, Yixiang Wang^2^, Zhibin Wang^1,2^*

1. Center for Cancer Immunotherapy of Institute of Biomedicine and Biotechnology, Shenzhen Institutes of Advanced Technology, Chinese Academy of Sciences, Shenzhen, Guangdong, PR China.
2. Department of Biopharmaceutical Sciences, Faculty of Pharmaceutical Sciences, Shenzhen University of Advanced Technology, Shenzhen, Guangdong, PR China;
3. University of Chinese Academy of Sciences, Beijing, China
4. Department of Biomedical Engineering, Southern University of Science and Technology, Shenzhen, Guangdong, PR China

*Corresponding authors:

Zhibin Wang Email: wangzhibin@suat-sz.edu.cn

**Supplementary material**

**Figure S1**

**
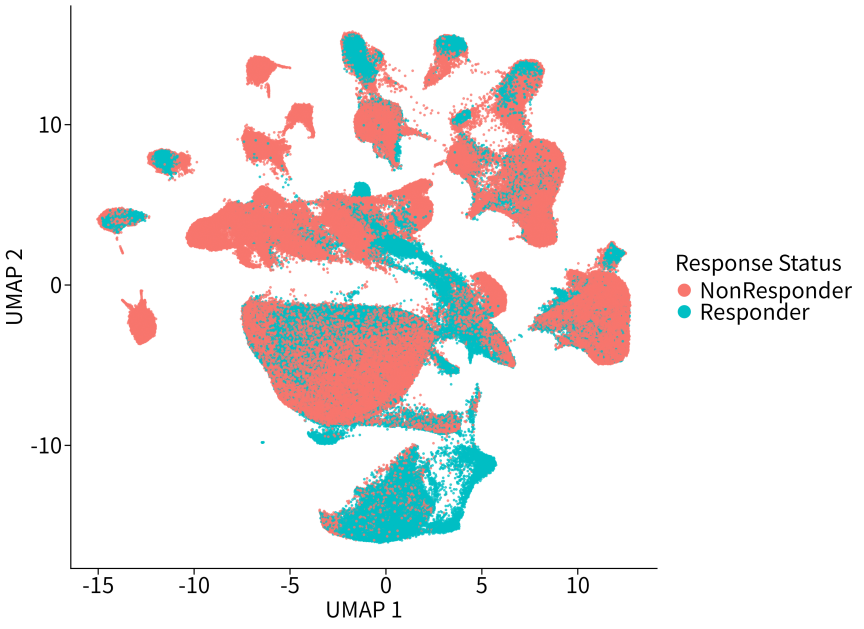
**

Figure S1. Distribution of Responder vs. Non-responder for all cells in UMAP space.

**Figure S2**

**
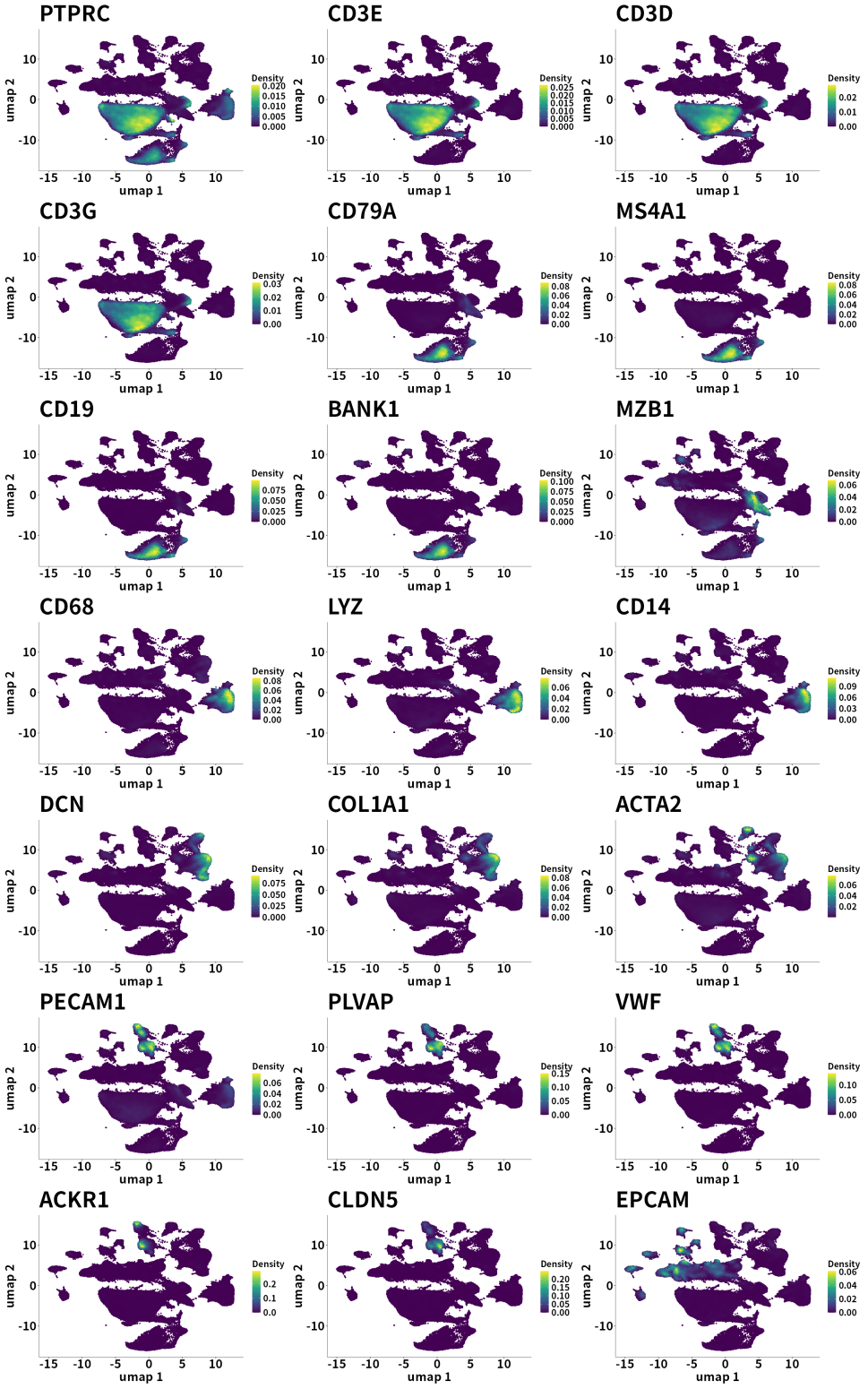
**

Figure S2. UMAP of all cells, colored by expression of canonical marker genes.

**Figure S3**

**
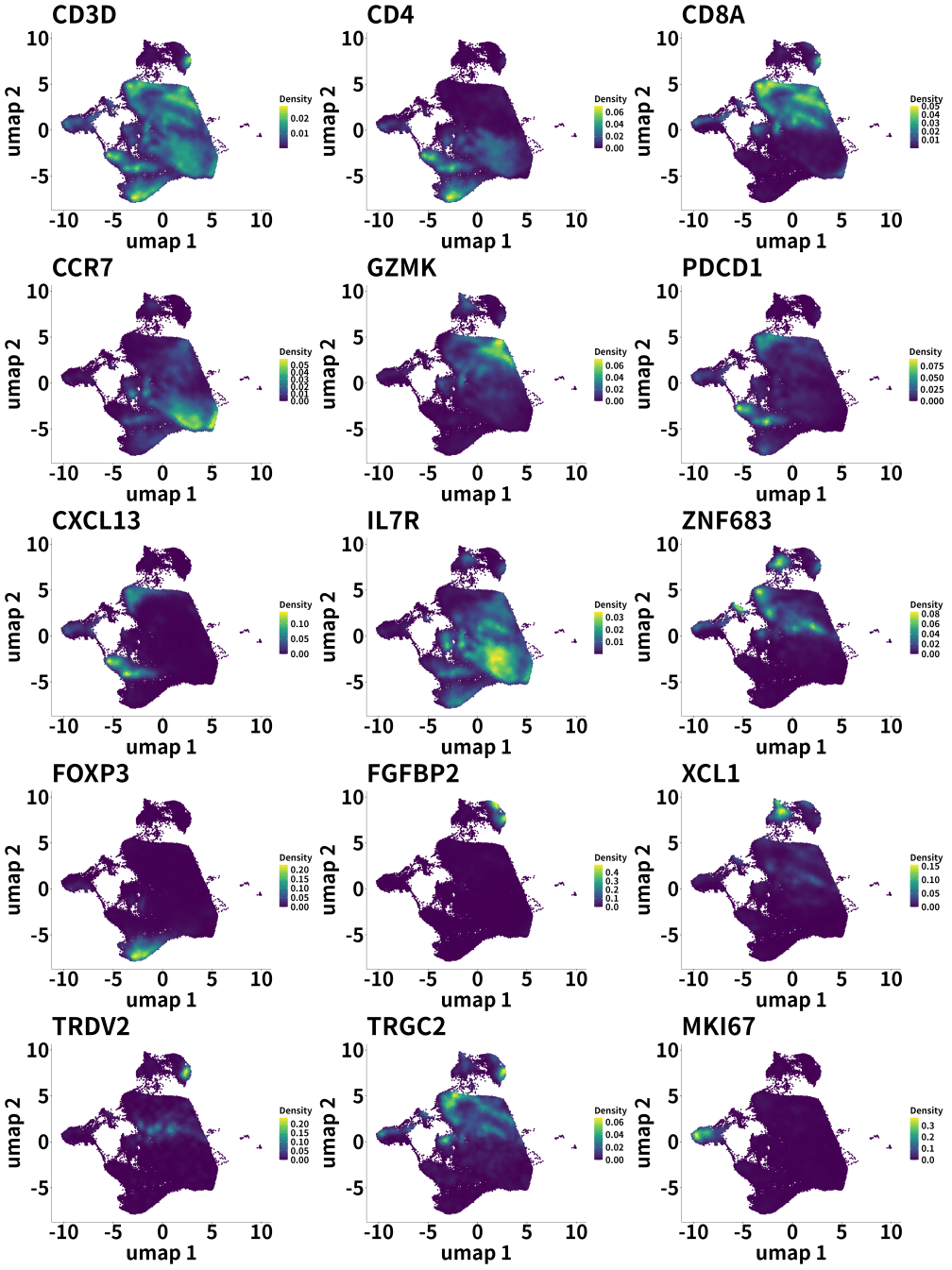
**

Figure S3. UMAP of T cells color-coded for one marker gene per T cell phenotype.

**Figure S4**

**
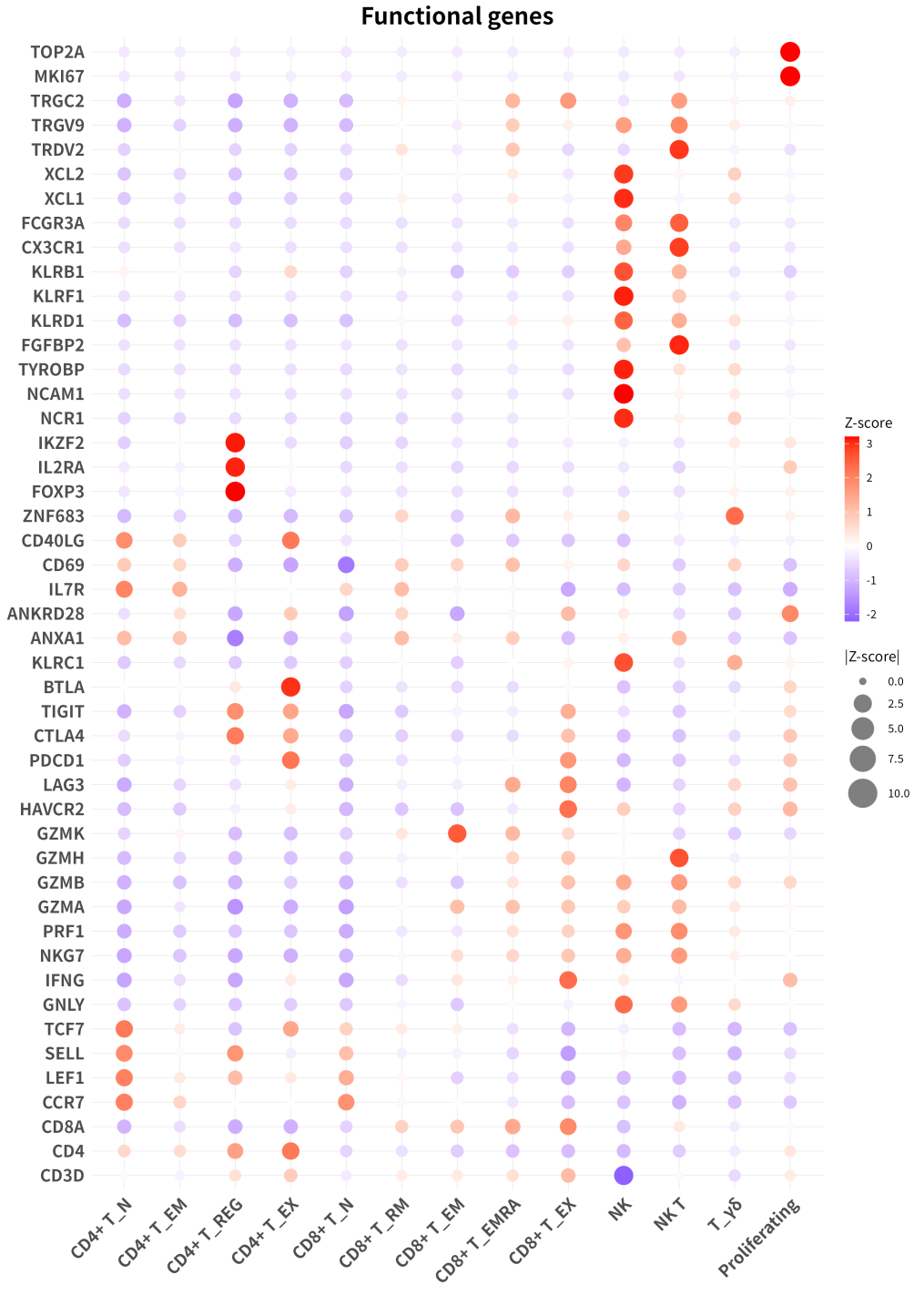
**

Figure S4. Heatmap of functional marker gene expression across T cell subtypes.

**Figure S5**

**
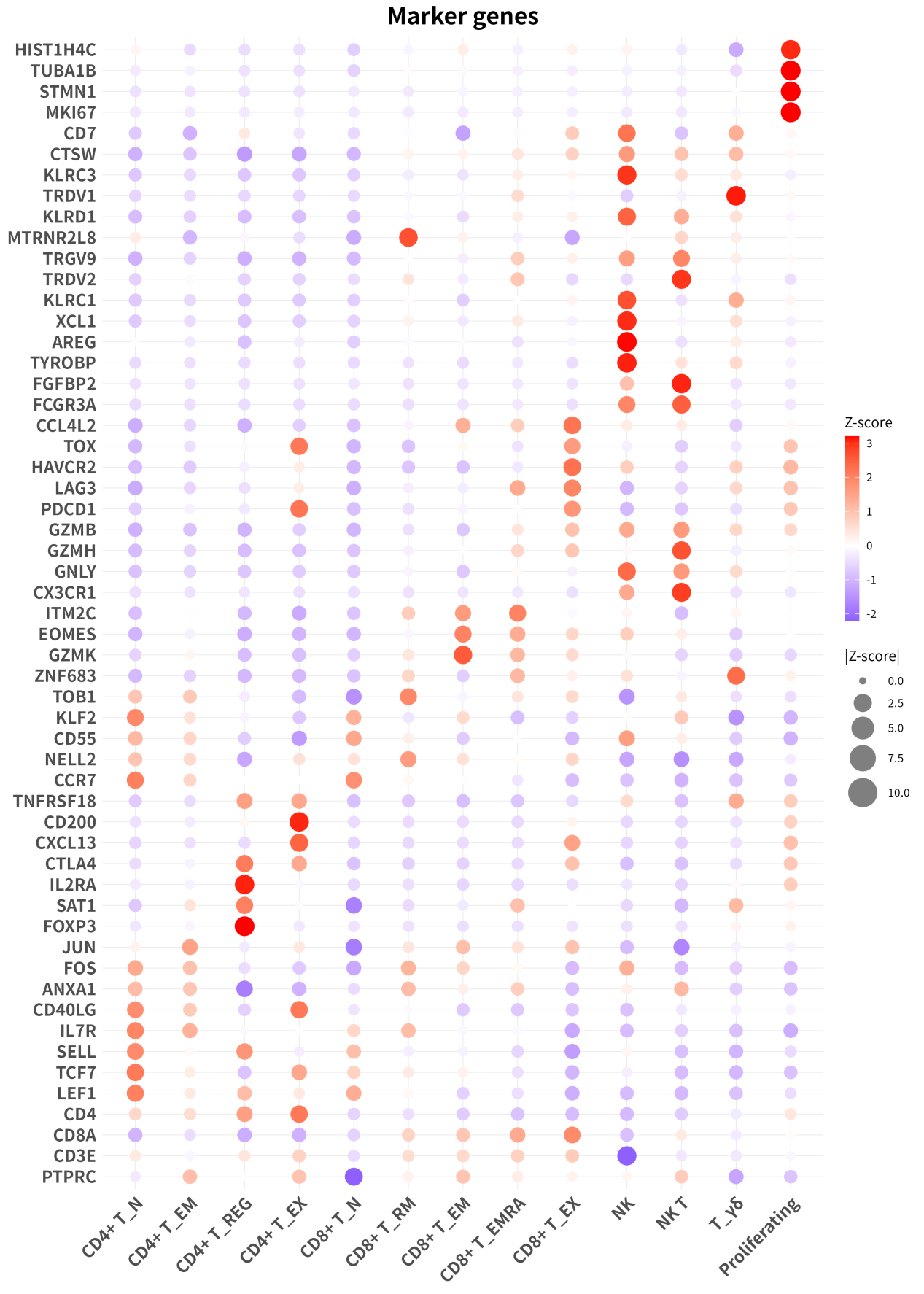
**

Figure S5. Heatmap of marker gene expression across T cell subtypes.

**Figure S6**

**
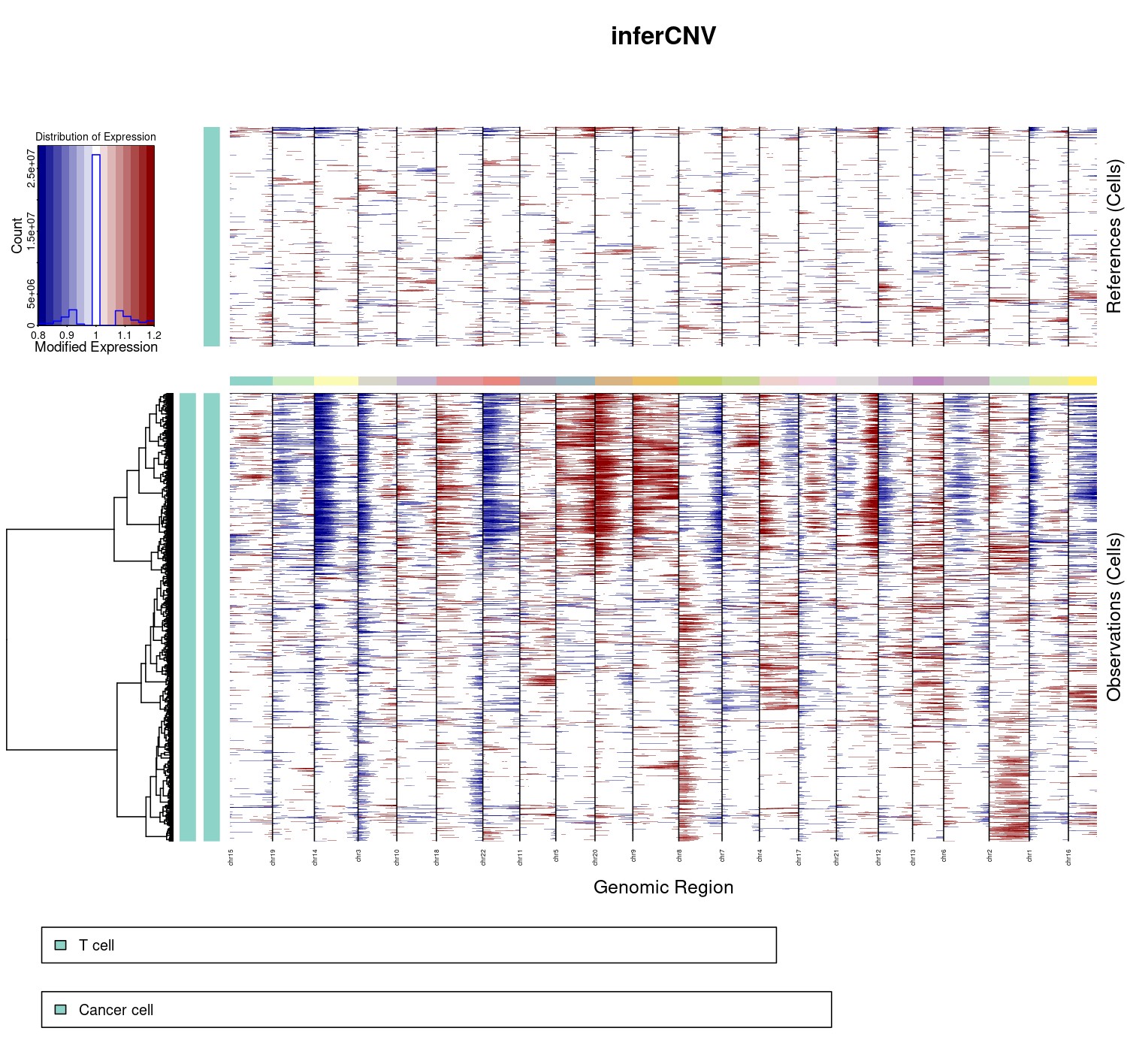
**

Figure S6. CNV profile in cancer versus T cells assessed using InferCNV based on scRNA-seq

**Figure S7**

**
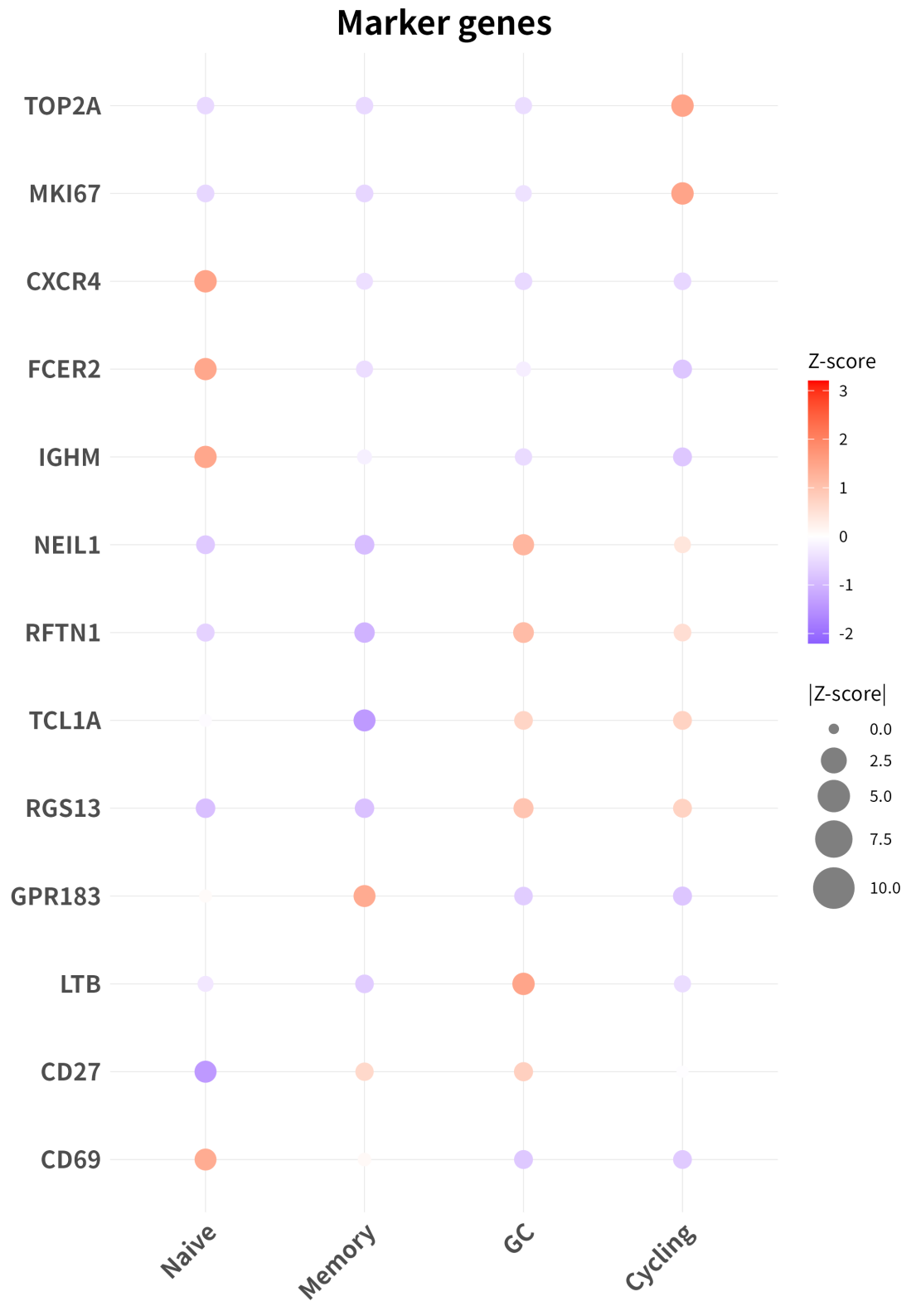
**

Figure S7. Heatmap of marker gene expression across B cell subtypes.

**Figure S8**

**
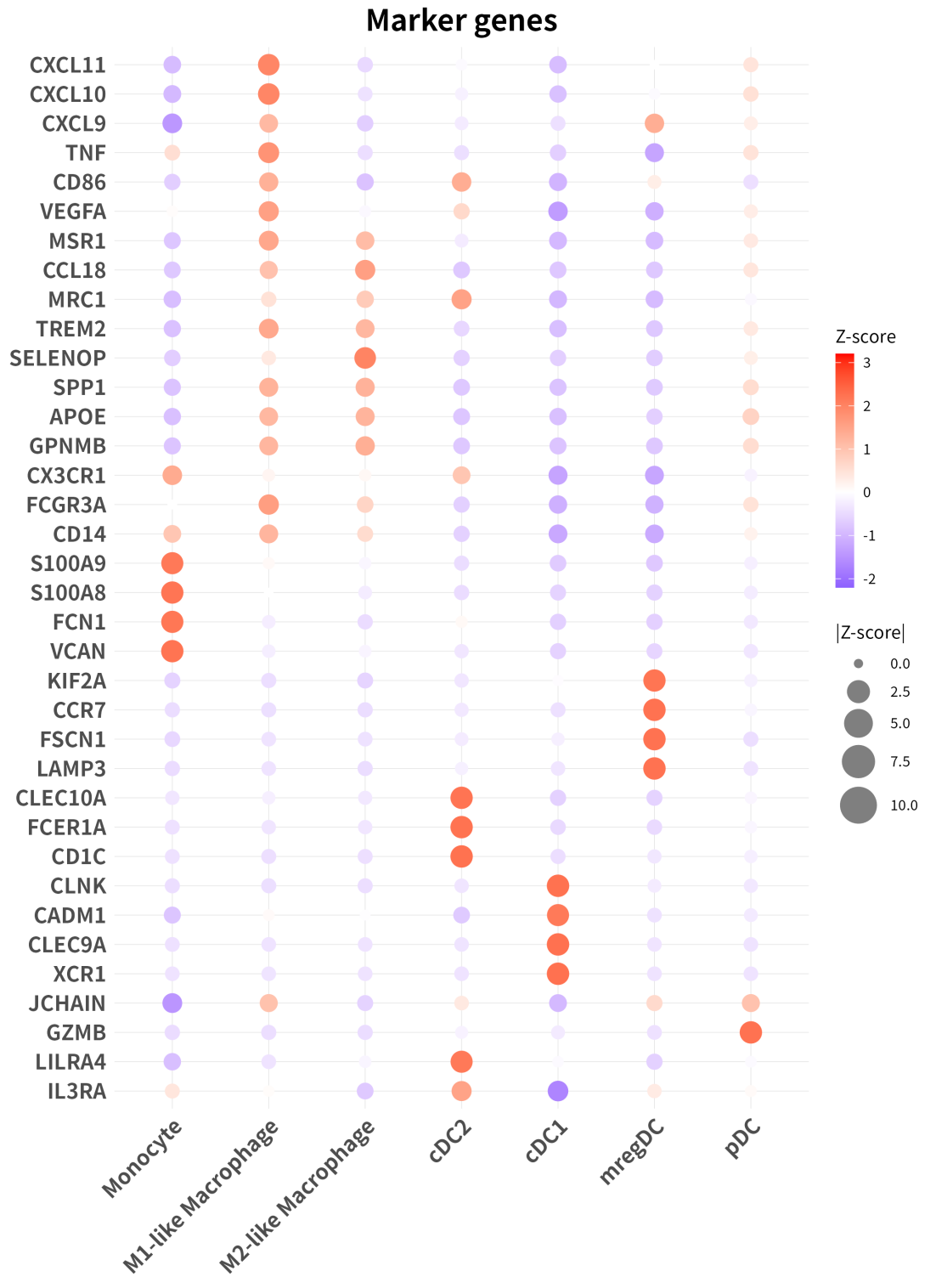
**

Figure S8. Heatmap of marker gene expression across Myeloid cell subtypes.

**Figure S9**


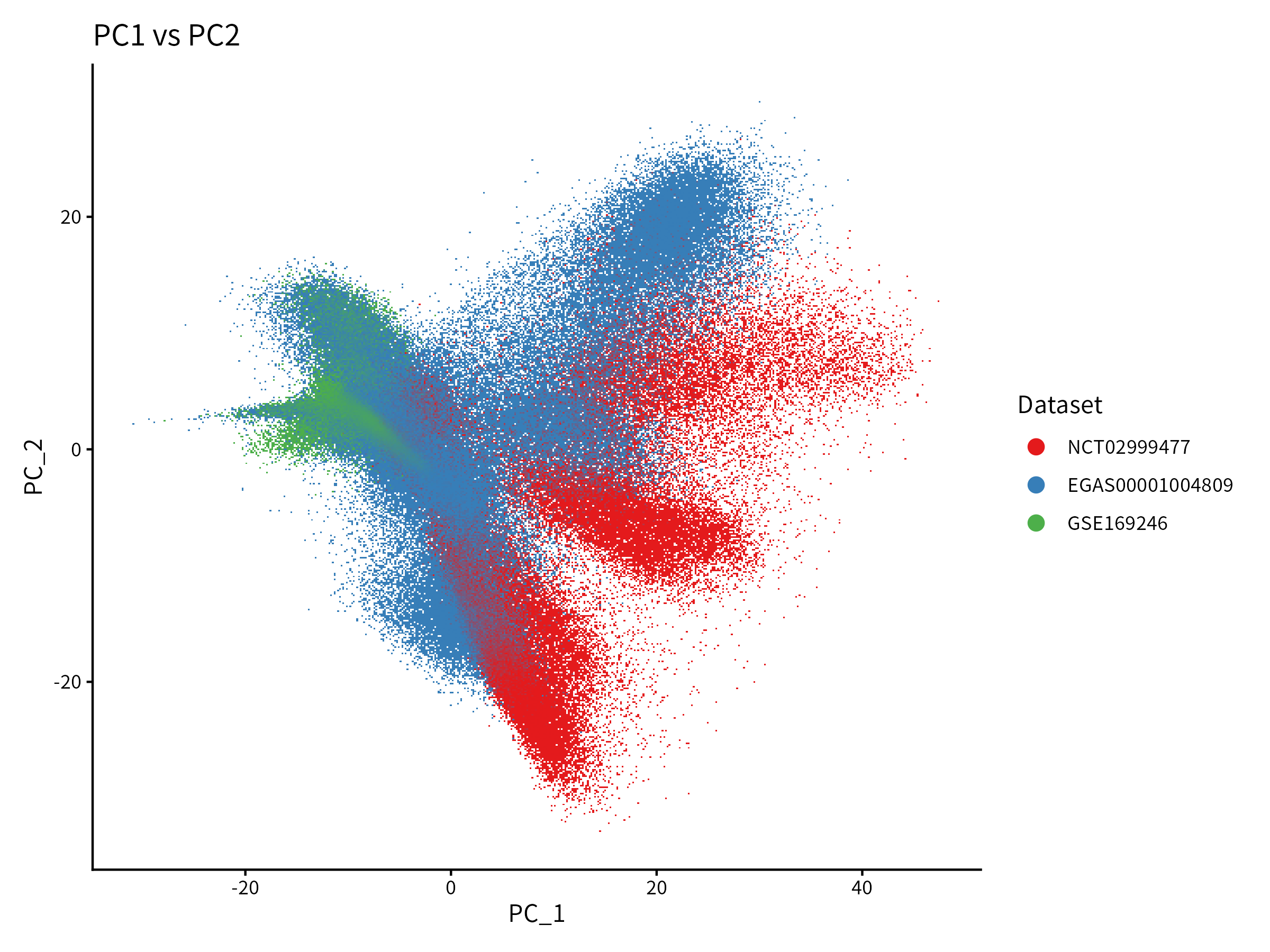


Figure S9. The batch-colored PCA plot after integration

**Figure S10**


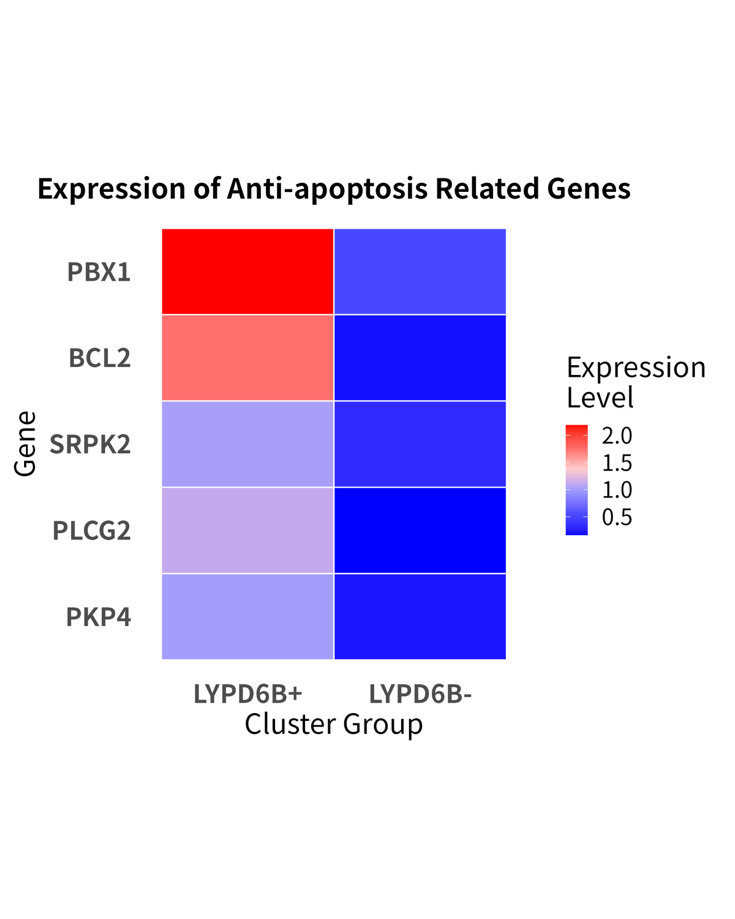


Figure S10. Heatmap of Anti-apoptosis Related Gene Expression in LYPD6B^+^ vs. LYPD6B^–^ Clusters

Table S1. Aboreviation and Full name.

| Aboreviation | Full name |
| --- | --- |
| TCGA-ACC | Adrenocortical carcinoma |
| TCGA-BLCA | Bladder Urothelial Carcinoma |
| TCGA-BRCA | Breast invasive carcinoma |
| TCGA-CESC | Cervical squamous cell carcinoma and endocervical adenocarcinoma |
| TCGA-CHOL | Cholangiocarcinoma |
| TCGA-COAD | Colon adenocarcinoma |
| TCGA-COADREAD | Colon adenocarcinoma/Rectum adenocarcinoma Esophageal carcinoma |
| TCGA-DLBC | Lymphoid Neoplasm Diffuse Large B-cell Lymphoma |
| TCGA-ESCA | Esophageal carcinoma |
| TCGA-FPPP | FFPE Pilot Phase II |
| TCGA-GBM | Glioblastoma multiforme |
| TCGA-GBMLGG | Glioma |
| TCGA-HNSC | Head and Neck squamous cell carcinoma |
| TCGA-KICH | Kidney Chromophobe |
| TCGA-KIPAN | Pan-kidney cohort (KICH+KIRC+KIRP) |
| TCGA-KIRC | Kidney renal clear cell carcinoma |
| TCGA-KIRP | Kidney renal papillary cell carcinoma |
| TCGA-LAML | Acute Myeloid Leukemia |
| TCGA-LGG | Brain Lower Grade Glioma |
| TCGA-LIHC | Liver hepatocellular carcinoma |
| TCGA-LUAD | Lung adenocarcinoma |
| TCGA-LUSC | Lung squamous cell carcinoma |
| TCGA-MESO | Mesothelioma |
| TCGA-OV | Ovarian serous cystadenocarcinoma |
| TCGA-PAAD | Pancreatic adenocarcinoma |
| TCGA-PCPG | Pheochromocytoma and Paraganglioma |
| TCGA-PRAD | Prostate adenocarcinoma |
| TCGA-READ | Rectum adenocarcinoma |
| TCGA-SARC | Sarcoma |
| TCGA-STAD | Stomach adenocarcinoma |
| TCGA-SKCM | Skin Cutaneous Melanoma |
| TCGA-STES | Stomach and Esophageal carcinoma |
| TCGA-TGCT | Testicular Germ Cell Tumors |
| TCGA-THCA | Thyroid carcinoma |
| TCGA-THYM | Thymoma |
| TCGA-UCEC | Uterine Corpus Endometrial Carcinoma |
| TCGA-UCS | Uterine Carcinosarcoma |
| TCGA-UVM | Uveal Melanoma |
| TARGET-OS | Osteosarcoma |
| TARGET-ALL | Acute Lymphoblastic Leukemia |
| TARGET-NB | Neuroblastoma |
| TARGET-WT | High-Risk Wilms Tumor |

Table S2. Summarized pathological and quantification data of immunohistochemical analysis of TOX1 in normal breast.

| no | Patient ID | Age | Tissue  type | Breast tissue type | Staining | Intensity | Quantity | Localization |
| --- | --- | --- | --- | --- | --- | --- | --- | --- |
| 1 | 2733 | 22 | Normal  tissue | Adipocytes | Not  detected | Negative | None | None |
|  |  |  |  | Glandular cell | Low | Weak | 75%-25% | Nuclear |
|  |  |  |  | Myoepithelial  cell | Not  detected | Negative | None | None |
| 1 | 3286 | 27 | Normal  tissue | Adipocytes | Not  detected | Negative | None | None |
|  |  |  |  | Glandular cell | Low | Weak | 75%-25% | Nuclear |
|  |  |  |  | Myoepithelial  cell | Not  detected | Negative | None | None |
| 1 | 3544 | 45 | Normal  tissue | Adipocytes | Not  detected | Negative | None | None |
|  |  |  |  | Glandular cell | Low | Weak | 75%-25% | Nuclear |
|  |  |  |  | Myoepithelial  cell | Not  detected | Negative | None | None |

Table S3: Summarized pathological and quantification data of immunohistochemical analysis of LYPD6B in breast cancer.

| **no** | **Patient ID** | **Age** | **Type** | **Staining** | **Intensity** | **Quantity** | **Localization** | **LYPD6B staining**  **observed in**  **tumor**  **infiltrating**  **cells** |
| --- | --- | --- | --- | --- | --- | --- | --- | --- |
| 1 | 1458 | 41 | Ductal  carcinoma | Low | Weak | >75% | Nuclear | N |
| 2 | 1775 | 55 | Duct carcinoma | Not detected | Negative | None | None | N |
|  |  |  |  | Not detected | Negative | None | None | N |
| 3 | 1874 | 80 | Duct carcinoma | Low | Weak | 75%-25% | Nuclear | N |
|  |  |  |  | Low | Weak | 75%-25% | Nuclear | N |
| 4 | 1875 | 72 | Duct carcinoma | Low | Weak | 75%-25% | Nuclear | N |
|  |  |  |  | Low | Weak | 75%-25% | Nuclear | Y |
| 5 | 1910 | 61 | Duct carcinoma | Medium | Moderate | >75% | Nuclear | N |
|  |  |  |  | Medium | Moderate | >75% | Nuclear | N |
| 6 | 2160 | 83 | Duct carcinoma | Not  detected | Negative | None | None | N |
|  |  |  |  | Not  detected | Negative | None | None | N |
| 7 | 2428 | 75 | Duct carcinoma | Low | Weak | >75% | Nuclear | N |
|  |  |  |  | Low | Weak | >75% | Nuclear | N |
| 8 | 2565 | 51 | Lobular  carcinoma | Medium | Moderate | >75% | Nuclear | N |
|  |  |  |  | Medium | Moderate | >75% | Nuclear | Y |
| 9 | 2805 | 59 | Lobular  carcinoma | Not  detected | Negative | None | None | N |
|  |  |  |  | Not  detected | Negative | None | None | N |
| 10 | 3546 | 58 | Lobular  carcinoma | Not  detected | Negative | None | None | N |
| 11 | 4193 | 43 | Lobular  carcinoma | Low | Weak | >75% | Nuclear | N |
|  |  |  |  | Low | Weak | >75% | Nuclear | N |

Table S4. Top 10 drug molecules ranked by docking score

| Drug | docking score |
| --- | --- |
| Venetoclax_(ABT-199) | -10.12 |
| Hypericin | -9.818 |
| Hypericin | -9.702 |
| Revaprazan_Hydrochloride | -9.688 |
| Venetoclax_(ABT-199) | -9.669 |
| Venetoclax_(ABT-199) | -9.605 |
| Evans_Blue | -9.583 |
| umbralisib_(TGR-1202) | -9.529 |
| Irinotecan_hydrochloride | -9.506 |
| Evans_Blue | -9.501 |
| Venetoclax_(ABT-199) | -9.452 |
| Venetoclax_(ABT-199) | -9.424 |
| Irinotecan_(CPT-11)_HCl_Trihydrate | -9.417 |
| Paritaprevir_(ABT-450) | -9.349 |
| Venetoclax_(ABT-199) | -9.348 |
| Venetoclax_(ABT-199) | -9.34 |
| Diammonium_Glycyrrhizinate | -9.328 |
| Evans_Blue | -9.328 |
| Evans_Blue | -9.322 |
| Flumatinib_(HH-GV-678) | -9.291 |
